# Acquisition and Evolution of Plant Pathogenesis–Associated Gene Clusters and Candidate Determinants of Tissue-Specificity in *Xanthomonas*

**DOI:** 10.1371/journal.pone.0003828

**Published:** 2008-11-27

**Authors:** Hong Lu, Prabhu Patil, Marie-Anne Van Sluys, Frank F. White, Robert P. Ryan, J. Maxwell Dow, Pablo Rabinowicz, Steven L. Salzberg, Jan E. Leach, Ramesh Sonti, Volker Brendel, Adam J. Bogdanove

**Affiliations:** 1 Department of Genetics Development and Cell Biology, Iowa State University, Ames, Iowa, United States of America; 2 Centre for Cellular and Molecular Biology, Council of Scientific and Industrial Research, Hyderabad, India; 3 Departamento de Botânica, IB-USP, Sao Paulo, Sao Paulo, Brazil; 4 Department of Plant Pathology, Kansas State University, Manhattan, Kansas, United States of America; 5 BIOMERIT Research Centre, BioSciences Institute, University College Cork, Cork, Ireland; 6 The Institute for Genomic Research, Rockville, Maryland, United States of America; 7 Institute for Genome Sciences, University of Maryland, Baltimore, Maryland, United States of America; 8 Center for Bioinformatics and Computational Biology, University of Maryland, College Park, Maryland, United States of America; 9 Department of Bioagricultural Sciences and Pest Management, Colorado State University, Fort Collins, Colorado, United States of America; 10 Department of Statistics, Iowa State University, Ames, Iowa, United States of America; 11 Department of Plant Pathology, Iowa State University, Ames, Iowa, United States of America; Research Institute for Children and the Louisiana State University Health Sciences Center, United States of America

## Abstract

**Background:**

*Xanthomonas* is a large genus of plant-associated and plant-pathogenic bacteria. Collectively, members cause diseases on over 392 plant species. Individually, they exhibit marked host- and tissue-specificity. The determinants of this specificity are unknown.

**Methodology/Principal Findings:**

To assess potential contributions to host- and tissue-specificity, pathogenesis-associated gene clusters were compared across genomes of eight *Xanthomonas* strains representing vascular or non-vascular pathogens of rice, brassicas, pepper and tomato, and citrus. The *gum* cluster for extracellular polysaccharide is conserved except for *gumN* and sequences downstream. The *xcs* and *xps* clusters for type II secretion are conserved, except in the rice pathogens, in which *xcs* is missing. In the otherwise conserved *hrp* cluster, sequences flanking the core genes for type III secretion vary with respect to insertion sequence element and putative effector gene content. Variation at the *rpf* (*r*egulation of *p*athogenicity *f*actors) cluster is more pronounced, though genes with established functional relevance are conserved. A cluster for synthesis of lipopolysaccharide varies highly, suggesting multiple horizontal gene transfers and reassortments, but this variation does not correlate with host- or tissue-specificity. Phylogenetic trees based on amino acid alignments of *gum*, *xps*, *xcs*, *hrp*, and *rpf* cluster products generally reflect strain phylogeny. However, amino acid residues at four positions correlate with tissue specificity, revealing *hpaA* and *xpsD* as candidate determinants. Examination of genome sequences of xanthomonads *Xylella fastidiosa* and *Stenotrophomonas maltophilia* revealed that the *hrp*, *gum*, and *xcs* clusters are recent acquisitions in the *Xanthomonas* lineage.

**Conclusions/Significance:**

Our results provide insight into the ancestral *Xanthomonas* genome and indicate that differentiation with respect to host- and tissue-specificity involved not major modifications or wholesale exchange of clusters, but subtle changes in a small number of genes or in non-coding sequences, and/or differences outside the clusters, potentially among regulatory targets or secretory substrates.

## Introduction

Comparative genomics is a powerful approach to discovering genetic features of related bacteria that have been acquired, modified, or lost during adaptation to particular environmental niches. Identification of such features is a first step toward understanding gene functions relevant to the adaptation. Comparative genomics has been particularly fruitful in understanding adaptation involving pathogenic exploitation of eukaryotic hosts. For example, it has led to the isolation of specific gene clusters that enable different bacterial pathogens to infect humans [1]–[4]. It also has helped define or refine relationships among animal pathogens and provide clues to the evolution of pathogenesis or specific pathogenesis-related functions [for a review, see 5]. Few comparative genomics studies have been carried out on plant pathogenic bacteria. In 2002, Van Sluys et al. [6] identified nineteen genes (encoding conserved hypothetical genes, iron transporters, and cell-wall modifying enzymes) common to all sequenced plant-associated bacterial genomes available at the time. More recently, a comparative analysis of sequenced Enterobacteriaceae identified genes specific to the plant pathogen *Erwinia carotovora* [7]. Comparative genomics has also provided novel insight into the role of horizontal gene transfer in shaping genomes of plant pathogenic xanthomonads [8]–[10].

*Xanthomonas* is a large genus of Gram-negative, yellow-pigmented, plant-associated bacteria. Pathogenic species and pathovars (pathogenic varieties, pv.) within species show a high degree of host plant specificity and combined are known to cause diseases on nearly 400 plant hosts, including both eudicots and monocots [11]. Many exhibit tissue-specificity, invading either host xylem vessels or the interveinal mesophyll apoplast of their host. Thus, the genus is a compelling subject for comparative genomics, as such analyses should shed light on how this group of bacteria has adapted to exploit an extraordinary diversity of plant hosts and host tissues. Understanding pathogenic adaptations of *Xanthomonas* will foster the development of needed improvements in bacterial plant disease control and prevention.

The genus *Xanthomonas* resides at the base of the gamma subdivision of the proteobacteria. The current taxonomic status of the genus is based on analysis of 16S–23S rDNA intergenic spacer sequences [12] and a combination of molecular markers such as rep-PCR, AFLP and other fingerprints [13], [14]. Twenty DNA homology groups (species) have been distinguished, comprising 80 pathovars [14], [15]. A species can encompass pathovars that infect diverse plant hosts and/or exhibit different patterns of plant colonization. For instance, *Xanthomonas campestris* includes pathovars that (collectively) infect different brassicaceous, solanaceous, and other plant species, and *Xanthomonas oryzae*, a species specific to rice and some wild relatives, comprises pathovars that either invade through the vascular system (*X. oryzae* pv. *oryzae*) or colonize the intercellular spaces of the parenchyma tissue (*X . oryzae* pv. *oryzicola*) [16]. Like *X. oryzae*, the *X. campestris* group also includes vascular and non-vascular colonizers [11], [17].

Complete genome sequences of six *Xanthomonas* strains had been published at the commencement of the present study. These are strains ATCC33913 and 8004 of *X. campestris* pv. campestris (XccA and Xcc8, respectively), a vascular pathogen of cabbage and other brassicas, including the model plant *Arabidopsis thaliana*; strain 306 of *X. axonopodis* pv. citri (Xac), the causal agent of citrus canker, a non-vascular disease; strain 85-10 of *X. axonopodis* pv. vesicatoria (Xav; formerly *X. campestris* pv. vesicatoria), a non-vascular pathogen that causes leaf spot on pepper and tomato; and strains KACC10331 and MAFF311018 of *X. oryzae* pv. oryzae (XooK and XooM, respectively), the vascular pathogen of rice [18]–[22]. During the course of this study, we finished and deposited in a public database the genome sequences of strain 756C [23] of *X. campestris* pv. armoraciae (Xca), a non-vascular pathogen with a host range similar to that of Xcc, and strain BLS256 [24] of *X. oryzae* pv. oryzicola (Xoc), the non-vascular counterpart of Xoo (AB et al., unpublished). We used these eight genome sequences in the analyses presented here. Subsequently, we completed the genome sequence of a third Xoo strain, PXO99^A^, and Vorholter et al. have recently published the genome sequence of a third Xcc strain, B100 [25], [26]. Complete or near complete genome data are also available for representatives of the closely related xanthomonads, *Xylella fastidiosa* (Xf) [27]–[30] and *Stenotrophomonas maltophilia* (Sma) [31, The Joint Genome Institute, US Dept of Energy, Genbank accession AAVZ00000000]. Xf is a group of fastidious, xylem-limited and insect-vectored plant pathogens with genomes roughly half the size of a typical *Xanthomonas* genome. Xf strains collectively cause disease on diverse hosts, with some specificity [32]. Sma is a non-plant pathogenic species that includes free-living as well as endophytic isolates and opportunistic human pathogens [33].

The *Xanthomonas* genome sequences we examined represent plant pathogens that are closely related but distinct in their host and tissue-specificity and that include paired vascular and non-vascular pathogens (Xcc and Xca, and Xoo and Xoc, respectively) of the leading models for plant biology, *A. thaliana* and rice. Furthermore, the sequenced *Xanthomonas* strains span three of the 20 homology groups (species) defined by Rademaker et al. [14], providing good representation of the genus as a whole. Our objective was to determine whether differentiation of species and pathovars with respect to host- and tissue-specificity is reflected across genomes in content and structure of several gene clusters that are known to be involved in pathogenesis in *Xanthomonas spp.* or are implicated in pathogenesis based on functions of homologous gene clusters in other pathogenic bacterial species.

## Results and Discussion

### *Xanthomonas* genomes and gene clusters examined

The *Xanthomonas* genome sequences examined are given in **Table 1**, grouped by strain host- and tissue-specificity. The general features of the genomes are similar (**Table S1**). Each includes a circular chromosome of approximately 5 Mb. The Xav genome includes four plasmids, and the Xac genome two. Average G+C content ranges from 63.6% (XooK and XooM) to 65.3% (Xca). The percent of genome that is predicted coding sequence ranges from 83.9% (XooM) to 90.3% (Xac). The number of predicted genes ranges from 4,598 (Xca) to 5,832 (XccA). Each genome harbors two ribosomal RNA operons.

**Table 1:** Classification of the examined *Xanthomonas* strains by host- and tissue-specificity^a^.

	Vascular	Non-Vascular
**Monocot**	XooK, XooM	Xoc
**Dicot**	Xcc8, XccA	Xca, Xac, Xav

a Abbreviations are as in the text.

We examined the *gum* gene cluster for extracellular polysaccharide synthesis [34], [35], the *xps* and *xcs* gene clusters for type II secretion [19], [36], the *hrp* gene cluster for type III secretion [37], the *rpf* gene cluster for regulation of pathogenicity factors [38], and an unnamed gene cluster involved in synthesis of lipopolysaccharide, which we hereafter refer to as the LPS gene cluster [39], [40] (the coordinates of the clusters in each genome are given in **Table S2**). For each cluster, we sought correlations of gene content and structure with host- and tissue-specificity, and we examined phylogenetic relationships by comparing concatenated sequences of the deduced gene products within the cluster across genomes.

### The *gum* gene cluster

The ability to produce capsular extracellular polysaccharide (EPS) is correlated with virulence in several plant pathogenic bacteria [41], and the importance of EPS to pathogenicity in *Xanthomonas* has been demonstrated with EPS-deficient mutant strains of Xoo, Xcc, and Xac [35], [42], [43]. EPS is important in biofilm formation and epiphytic fitness [43], [44]. It is postulated to promote colonization of plant tissues and to provide protection from harsh environmental conditions, and it contributes to occlusion of vascular elements in wilts and blights [41], [45]. Synthesis of the *Xanthomonas* capsular EPS, xanthan, is carried out primarily by the twelve products of the roughly 16 kb *gumB-gumM* operon [34], [35]. Additional open reading frames (ORFs) designated as *gum* genes, *gumA* and *gumN*, *-O*, and *-P*, reside up and downstream (respectively) of *gumB-gumM* [34], but a role for these genes in xanthan biosynthesis has not been demonstrated. Recently, *gumN* and an intervening ORF were shown to be co-transcribed with *gumB*-*gumM* in Xoo, but *gumA* is clearly in a distinct operon [46].

**Figure 1:**
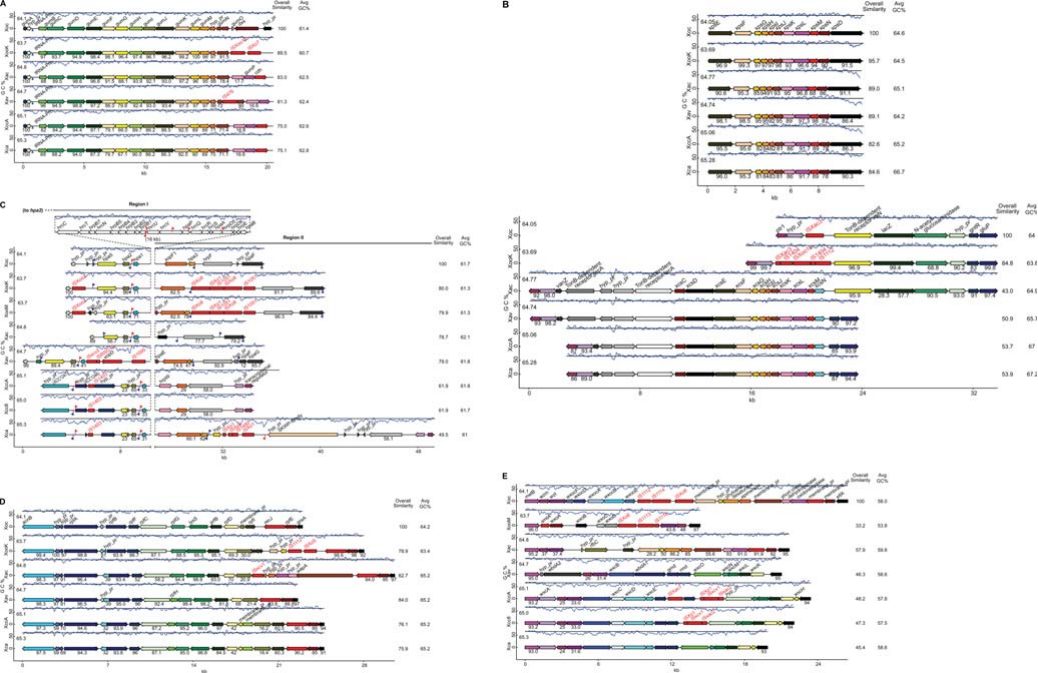
Comparison of six clusters of genes involved or implicated in pathogenesis among *Xanthomonas* strains representing three species and six pathovars. Sequences of *X. oryzae* pv. oryzicola strain BLS256 (Xoc), *Xanthomonas oryzae* pv. oryzae KACC100331 (XooK) and MAFF311018 (XooM), *X. axonopodis citri* strain 306 (Xac), *X. axonopodis* pv. vesicatoria strain 85-10 (Xav), *X. campestris* pv. campestris strains ATCC33913 (XccA) and 8004 (Xcc8), and *X. campestris* pv. armoraciae strain 756C (Xca) were used. Arrows represent individual genes. For each cluster across genomes, homologs are shown in like colors. Gene identities are given (non-redundantly) above each gene. The blue trace above each cluster represents GC content (window size: 160 bp, step: 40 bp). The black line above each cluster marks the average GC content of the genome, specified below and to the left of the line. Shown for each gene is the percent identity of the predicted product to that of the corresponding gene (if present) in the genome shown at the top. The overall similarity of each cluster to the cluster at the top is given at the near right. The average GC content of each cluster is given at the far right. Where clusters from different strains of the same pathovar are essentially identical, only one is represented. Insertion sequence elements are indicated by red rectangles, tRNA genes by blue triangles and plant-inducible promoter sequences (PIP boxes) by red or blue flags. A red flag represents a perfect PIP box and a blue flag represents an imperfect one. The orientation of the PIP box is represented by the orientation of the flag above or below the cluster. A, the *gum* gene cluster; B, the *xps* and *xcs* gene clusters; C, the *hrp* gene cluster; D, the *rpf* gene cluster; E, the lipopolysaccharide biosynthesis gene cluster bordered by the *etfA* and *metB* genes.

The nucleotide sequences of the *gum* cluster, delimited by and including *gumA* and *gumN*, and approximately 4 kb of sequence downstream of *gumN*, were retrieved from each genome and compared (**Figure 1A**). The cluster is highly conserved with respect to overall gene content and order, including the ORF between *gumM* and *gumN*. Differences among strains are limited to insertion sequence (IS) elements in or near *gumN* and differential content of genes outside the core cluster, including *gumO*, *gumP*, and *chd2*. None of these genes, however, have been shown to play a role in xanthan biosynthesis. (For a complete discussion of differences observed at the *gum* gene cluster, see **Text S1**.)

As noted by Lima et al. [10], the *gum* gene cluster has features of a pathogenicity island, including a lower than average G+C content and a flanking tRNA gene (**Figure 1A**). Indeed, the cluster is absent from the Sma genome sequences, suggesting that acquisition of *gum* genes was an important adaptation toward plant pathogenicity. Consistent with the notion that *gum* genes were acquired subsequent to the divergence of the *Xanthomonas* and *Stenotrophomonas* lineages, the regions flanking the *gum* locus in the *Xanthomonas* genomes are conserved and colinear in the Sma genomes, including the tRNA gene. A cluster containing *gumB* though *gumF* and *gumH* is present in the Xf genomes, but the genomic context is distinct, suggesting independent acquisition of these genes in the *Xylella* lineage. Interestingly, in the sugarcane pathogen *X. albilineans*, PCR failed to detect any of ten gum genes assayed, using primer sequences conserved across Xac, Xcc, and Xoo [47]. This species produces an exopolysaccharide structurally related to but distinct from xanthan and compositionally more similar to the exopolysaccharide produced by Xf [48], [49]. Production has only been observed in infected sugarcane stalks and appears to require plant components [50]. Direct comparison of the *X. albilineans* genes for exopolysaccharide production with the *gum* genes awaits completion of the first *X. albilineans* genome sequence, which is underway (P. Rott, personal communication). Nevertheless, the data available suggest that, as in Xf, these genes were acquired independently. The *gum* genes therefore, likely represent a relatively late adaptation in the lineage that gave rise to the *X. axonopodis*, *X.campestris*, and *X. oryzae* clades.

### The *xps* and *xcs* gene clusters

The type II secretion (T2S) system is the main terminal branch of the general secretory pathway in proteobacteria, mediating the transport of proteins into the extracellular space following their N-terminal signal peptide–dependent deposition into the periplasm. The T2S system was discovered in *Klebsiella oxytoca*, in which at least thirteen linked *pul* genes are required for secretion of the starch-hydrolyzing lipoprotein pullulanase [51], [52]. It has since been found important to the virulence of many animal and plant pathogens, including Xcc [36], [53] and Xoo [54], [55], exporting proteins such as toxins, proteases, lipases, and phospholipases, as well plant cell wall–degrading enzymes such as cellulases, pectinases and xylanases [56]. Two T2S system gene clusters, *xps* and *xcs* [19], are represented among the sequenced *Xanthomonas* strains (**Figure 1B**). The *xps* cluster consists of 11 genes in two predicted transcriptional units, the first of which contains genes *xpsE* and *xpsF* and the second *xpsG* through *xpsN* and *xpsD* [57]. The *xcs* cluster consists of one predicted operon containing 12 genes, *xcsC* through *xcsN* [57]. Corresponding *xps* and *xcs* gene names indicate homology, with the exception of *xpsN*, which is a homolog of *xcsC* [58]. No homolog of *xcsN* is present in the *xps* cluster. The *xps* cluster should not be confused with loci involved in synthesis of xanthan precursors, designated as xpsI, xpsII, *etc*. [59].

The *xps* cluster is present in all eight *Xanthomonas* genomes, as well as the Xf and Sma genomes. In contrast, *xcs* genes are present only in the Xac, Xav, Xcc, and Xca strains, each of which infects eudicots (**Figure 1B**). As noted previously [60], the *xcs* cluster sequences are more similar to the T2S gene cluster in *Caulobacter crescentus*, a member of the alpha subdivision of the proteobacteria, than to the *xps* cluster. The average G+C content of the cluster is also slightly above average for each genome (**Figure 1B**). Further highlighting the distinction, XpsE and XcsE belong to distinct T2S:E subfamilies, which differ by an N-terminal domain, N0, that is present and essential in XpsE [61], but missing from XcsE.

In strains with an *xcs* cluster (Xac, Xav, Xca, and Xcc), a TonB-dependent receptor (TBDR) gene and two hypothetical protein genes are located upstream of *xcsC* (leftward in the figure). In Xac and Xav only, beyond these genes is another TBDR gene. The region beyond that is again colinear across genomes, beginning with a homolog of the teicoplanin resistance gene *vanZ*, followed (to the left) by a hypothetical protein gene and the pteridine reductase gene *ptr1*. Downstream of *xcsN* in Xac are four genes (five ORFs, since the second gene is split by a frame shift), including another TBDR gene, that are absent from Xav and the *X. campestris* strains. Following the four-gene insertion/deletion, the genomes resume colinearity, starting with the *gntR* gene and (to the right) the glucose/galactose transporter gene *gluP*. Moreira et al. [60] reported that the three upstream and four downstream genes flanking the *xcs* cluster in Xac are conserved flanking the T2S genes in *C. crescentus* and that genes up- and downstream of these in Xac are conserved and linked in Xf, suggesting that the region constitutes an island that was inserted in *Xanthomonas* or deleted from *Xylella*. Specifically, we observed that the genes between the *vanZ* homolog and *gluP* are missing in Xf, replaced by a glucokinase gene and a short non-coding region. This arrangement is conserved in the Sma genomes except for the replacement of the non-coding region with an acetylhexosaminidase gene and a TBDR gene. This similarity suggests that the *Xylella* locus rather than the *Xanthomonas* locus more closely reflects the ancestral arrangement and that the *xcs* cluster is in fact an insertion in *Xanthomonas*. Consistent with this conclusion, the absence of the *xcs* cluster from the Xoc and Xoo genomes presents an arrangement distinct from that in *Xylella*. Colinearity of the Xoc and Xoo genomes with the region upstream of *xcsC* in the other *Xanthomonas* genomes exists, extending (from the left toward *xcsC*) up to but not including the *vanZ* homolog, which is missing. This region is followed by transposase genes and IS element sequences that are different between Xoc and Xoo. Thereafter, the Xoc and Xoo genomes are colinear with the Xac genome beginning with the TBDR gene immediately downstream of *xcsN* in Xac and extending through *gntR* (**Figure 1B**). The distinct endpoints of colinearity with the other genomes and differences in intervening gene content between the Xoc and Xoo vs. the Xf and Sma genomes (not shown) strongly suggest that the *xcs* cluster was present in but subsequently lost from the *X. oryzae* lineage.

Because several *xps* mutations that reduce virulence have been isolated in Xcc, the *xcs* genes, despite their similarity to *xps* counterparts, clearly are not functionally redundant. And, no mutations that affect virulence have been reported in the *xcs* cluster in any strain. The *xcs* cluster may play a role in processes not associated with pathogenesis, or in fact may be non-functional. Even presuming a role in pathogenesis, the fact that the Xf strains infect dicots, but lack the *xcs* cluster, argues against a host-specific role for these genes.

### The *hrp* gene cluster

The *hrp* (*h*ypersensitive *r*eaction and *p*athogenicity) gene cluster encodes components of the T3S system [62] and constitutes an important contributor to plant colonization by many plant pathogenic species. Individual genes have been classified and named as *hrp*, *hrp*-conserved (*hrc*), or *hrp*-associated (*hpa*). In the strains compared here, the cluster generally comprises 24 genes located in or adjacent to two designated subregions [63], the core *hrp* cluster (Region I), extending from *hpa2* to *hpaB*, and the *hrpF* peninsula (Region II), a more variable subregion centered on *hrpF* (**Figure 1C**). Originally, the designations “*hrp*” and “*hrc*” indicated loci that are required for induction of non-host hypersensitive reaction and for pathogenicity, and individual genes in the loci were given these designations. However, not all *hrp* and *hrc* genes have this phenotype. With some exceptions, the *hrp* gene sequences are unique to *Xanthomonas* and some other genera with related *hrp* clusters, while *hrc* genes are clearly conserved among the xanthomonads and many other pathogens of animals and plants [64]. *hpa* genes localize to the cluster and are important in pathogenicity to differing degrees, depending on the gene. Some have no known roles, some function in targeting type III secreted proteins to the secretion apparatus, and some are themselves secreted and in some cases translocated into host cells [63], [65]–[67]. For some *hrp*-associated genes whose products are secreted, names that reflect this fact have been adopted (e.g. *X*anthomonas *o*uter *p*rotein *F1* or *xopF1*).

The *hrp* cluster Region I and II sequences and structures support a model of monophyletic inheritance followed by fraying of the outer ends of the regions by insertions, deletions, and rearrangements (for details, see **Text S1**). The presence of remnants of several ORFs, including *xopF1* and *hpa5*, in most or all of the genomes also supports this model. Xca and the Xcc strains share the same left and right border sequences of Regions I and II, again reflecting the close relationship between these two pathovars. The remaining genomes share a common left border for Region I. At the same time, rearrangements have obscured the ancestral right boundaries of the *hrp* cluster (Region II) in all but the *X. campestris* strains. Ten kilobases of sequence immediately adjacent to the end of *hrpF* are unique to these strains. No evidence was found to indicate whether the left border sequences of the *X. campestris* strains or those in the Xav, Xoo, Xoc, and Xac genomes represent the ancestral border. All borders may have been the result of rearrangements after the divergence of the lines, and the ancestral borders may, in fact, have been deleted in all lines. Strain-specific genes in the left border of Region I or in Region II, *e.g. xopD* in Xav, or the candidate SKWP family effector gene in Xca, may represent pathovar-specific adaptations, but no clear general correlations of gene content with host tissue- or class- (monocot vs. eudicot) specificity are apparent.

The *hrp* gene cluster was previously identified as a pathogenicity island in *Xanthomonas* [68]. It is absent from the Xf and Sma genomes. Interestingly, it is missing also from *X. albilineans* and two other *Xanthomonas* spp. [69]. These three species form a distinct phylogenetic clade based on 16s rDNA sequence alignment [70]. Thus it seems likely that acquisition of the *hrp* gene cluster, like the *gum* cluster, was a relatively late adaptation in the lineage that led to the *Xanthomonas* strains in the present study. *X. albilineans* has a genome roughly two-thirds the size of the sequenced *Xanthomonas* genomes and depends largely on a single toxin for pathogenesis [71], [72]. The *X. albilineans* genome may represent a primitive genome that lacks many of the adaptations present in other *Xanthomonas* strains, or, as postulated for *Xylella* [73], it may represent a reduced and highly adapted genome with a minimal complement of genes needed for survival within a plant. Another possibility is that *X. albilineans* is an evolutionary intermediate between Xf and other *Xanthomonas spp*. [47]. Once a *X. albilineans* genome sequence is complete, comparisons with the genomes of other strains will shed light on this question as well as the question of whether *hrp* (and *gum*) genes were present in and then lost from the *X. albilineans* lineage, or were never introduced into it.

### The *rpf* gene cluster

The *rpf* gene cluster positively regulates the synthesis of extracellular enzymes, extracellular polysaccharide, biofilm dispersal and virulence in Xcc [38], [74]–[77]. Several of the Rpf proteins are involved in an intercellular signal-response system that links perception of the diffusible signal factor (DSF) *cis*-11-methyl-2-dodecenoic acid [78] to the regulation of virulence factor synthesis and biofilm dispersal. RpfB and RpfF direct the synthesis of DSF, whereas the hybrid sensor kinase RpfC and the HD-GYP domain regulator RpfG are implicated in DSF signal perception and signal transduction [74], [76], [79]. In Xcc, *rpfH* is transcribed as part of the *rpfGHC* operon, though the function of RpfH is unknown. Other *rpf* genes (*rpfADEI*) are not implicated in the DSF regulatory system and have minor regulatory roles in Xcc [75]. The DSF regulatory system is also implicated in virulence in other xanthomonads. Mutation of *rpfC* or *rpfF* in *Xoo* and *Xac* leads to loss of virulence on rice and citrus, respectively [76], [80]–[82], and disruption of *rpfG* reduces virulence in Xoc [83]. DSF has not been isolated from any of these strains but it is likely to be highly similar if not identical to DSF from Xcc. DSF from *X. fastidiosa* and *Burkholderia cenocepacia* are structurally only slightly different from Xcc DSF, and they are functionally conserved, inducing DSF-responsive reporter genes when added to cultures of DSF-deficient Xcc [84]–[86].

Significantly, all *rpf* genes with an established role in the DSF regulatory system in Xcc (*rpfBFCG*) are intact in all the *Xanthomonas* genomes (**Figure 1D**). This is true also of the Xf and Sma genomes, indicating that the *rpf* cluster is ancestral. In fact, the *rpfF* gene was recently shown to be important in Sma virulence and resistance to antibiotics [87]. RpfE is also conserved. As noted for the *hrp* cluster, minor, strain-specific differences in gene content exist in the *rpf* cluster, but correlations with host- or tissue-specificity are not readily apparent (for details, see **Text S1**).

### The LPS gene cluster

LPS is a component of the bacterial cell surface that comprises three covalently linked structures: an outer membrane–bound moiety called lipid A, a core oligosaccharide, and an outermost polysaccharide known as the O-chain [88]. Structural variations in LPS, in particular the O-chain (also “O-antigen”), often account for variations in serotype as well as the emergence of new virulent strains associated with epidemics of human and livestock disease [89], [90]. LPS has been implicated previously in plant pathogenesis owing to the isolation of reduced virulence mutants that exhibited LPS deficiencies [for a review, see 91]. Plants recognize LPS or LPS components as pathogen-associated molecular patterns (PAMPs) [92], which trigger innate defense responses [91], [93]–[95]. In the *Rhizobium*-legume symbiosis, structural changes in the O-chain take place during nodulation, suggesting an adaptive role [96]. A cluster of 15 genes in Xcc strain B100 governs the synthesis of the core and O-chain of LPS [40]. This locus has G+C content markedly lower than the average for the genome. In Xoo strain BXO1, the locus is substituted by a largely divergent and apparently non-homologous set of genes for LPS core and O-chain synthesis, also with atypical G+C content [39], [97]. Two other Xoo strains (BXO8 and Nepal624) and a Xoc strain (BXOR1) contain yet other distinct clusters, based on PCR and Southern hybridization results [97]. All clusters are flanked by the highly conserved *etfA* and *metB* genes [97]. Mutations at this locus in BXO8 (PP and RS, unpublished), in Xcc8 [18], and in Xoc [83] are associated with reductions in virulence.

Comparison of gene content between *etfA* and *metB* across the sequenced genomes revealed a remarkably high degree of variation both in number and in identity of genes (**Figure 1E**). The sizes of the clusters range from 14.4 kb (XooK and XooM) to 26.5 kb (Xoc). The number of genes varies from seven (the Xoo strains) to 15 (Xoc and the Xcc strains). The G+C content of each cluster is low compared to the average for each genome, ranging from 55% (in XooK) to 60.3% (in Xac). Five of the seven genes in the XooK and XooM cluster do not have orthologs in the Xoc cluster. The exceptions are *wzm* and *wzt*, which are predicted to encode components of an ABC transporter system for export of LPS. Interestingly, these genes exhibit only 48.2 and 41.7 % identity to their Xoc orthologs at the amino acid level. Similarly, though Xoc and the Xcc clusters all contain 15 ORFs, again, only *wzm* and *wzt* have orthologous counterparts, and these exhibit a low level of amino acid identity (25.1% and 31.6 %, respectively).

Some similarities exist across genomes. For example, in each genome the genes are organized in two convergent blocks, suggestive of operons. IS elements are located at the junction of these apparent transcriptional units in several genomes. The Xca, Xcc8, and XccA clusters are essentially identical, with the exception of IS elements in the Xcc strains and a single ORF substitution at the end of the putative transcriptional unit proximal to *etfA* in Xca. This half of the Xca cluster is identical to that of Xav, except that Xav is missing one gene, *wxocH*. The *metB* proximal part of the cluster is largely similar between Xav and the *X. campestris* strains, except for a substitution in Xav that replaces three genes, *wxcC*, *-D*, and *-E*, with one, *wbdA1*. Between Xac and Xoc, the *etfA* proximal half of the cluster is essentially identical. Outside of the cluster, upstream of *etfA*, a distinct region that contributes to LPS biosynthesis spans approximately 11 kb [98], [99]. This locus is highly conserved, differing only in Xac and Xav by the insertion of a UDP-glucose dehydrogenase–encoding gene roughly in the center (not shown).

Overall, there is no apparent correlation of the content of the LPS biosynthetic gene cluster between *etfA* and *metB* with host- or tissue-specificity. Indeed, though the cluster of the BXO1 strain of Xoo mentioned above is essentially identical to that of XooK and XooM, the cluster in Xoo strain BXO8 is similar to that of Xac [97], [100], and the cluster in the B100 strain of Xcc shows near 100% identity to that of Xca [100]. Also, the Xav and Xac clusters are distinct. Thus, interspecies, interpathovar, and even interstrain variation is evident, suggesting that changes at this locus have not been strictly coincident with differentiation of species and pathovars. Rather, this locus seems to have been under intense diversifying selection and subject to frequent exchanges mediated by horizontal transfer and recombination. The atypical G+C content of the locus in each genome is consistent with this deduction. Examination of the two Sma genomes indicates that variability at this locus is not unique to the *Xanthomonas* clade. The locus is 30 kb in strain K279a and only 15 kb in strain R551, with only four genes common to both. Forces driving variability as this locus are therefore not likely limited to interactions with plant hosts, but may include interactions with animal hosts or phage, or other environmental interactions.

### Relationships of gene clusters across strains relative to ribosomal RNA sequences

**Figure 2:**
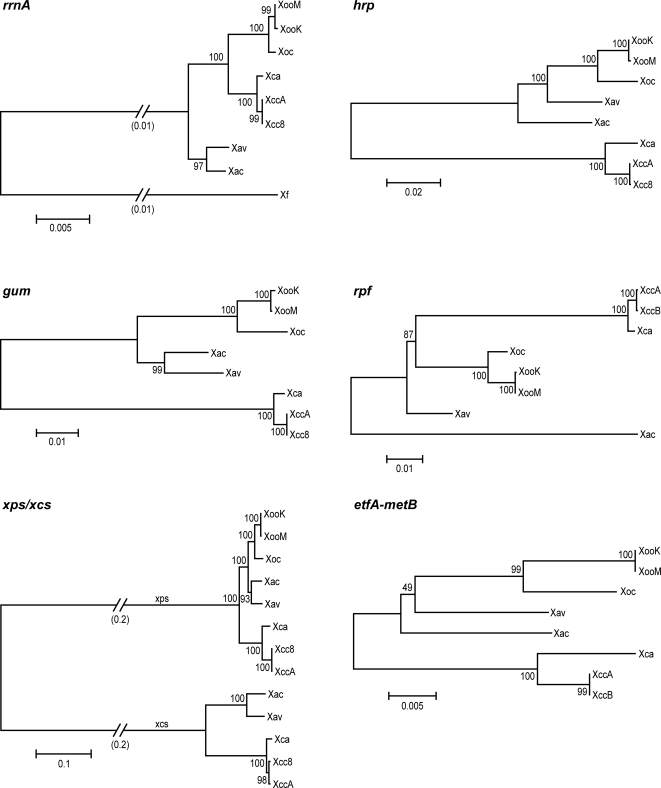
Relationships across *Xanthomonas* strains of ribosomal RNA sequences and sequences of pathogenesis-associated gene clusters. Phylogenetic trees generated as described in Materials and Methods are shown. *rrnA*, the *rrnA* operon (nucleotide alignment); *gum*, GumB through GumJ; *xps/xcs*, XpsD plus XpsE through XpsN, and XcsD through XpsM plus XpsC; *hrp*, HpaB through Hpa2; *rpf*, AcnB through RpfD; *etfA-metB*, EtfA and MetB, which flank the LPS biosynthetic locus (see Figure 1E). Strain abbreviations are as in the text. Sequence from *Xylella fastidiosa* 9a–5c was used to root the *rrnA* tree. Numbers above and below branch points are bootstrap values (as percent) for neighbor-joining with 1000 replicates. Scale represents relative distance as a function of substitutions over time.

To assess the extent to which relatedness of cluster sequences across strains reflects shared host-or tissue-specificity, a phylogenetic tree for each cluster was built based on alignments of concatenated sequences of the predicted gene products of that cluster (**Figure 2**). Thus, for the *gum* gene cluster, the amino acid sequences of GumB through GumJ in each strain were joined end to end, and the joined sequences from each strain aligned to one another. The *xcs* and *xps* gene clusters were analyzed together, using concatenated sequences of XcsC through XcsM, and of XpsN (the ortholog of XcsC) and XpsD with XpsE through XpsM. For the *hrp* gene cluster, HpaB through Hpa2 were concatenated and aligned. For the *rpf* cluster, sequences of AcnB through RpfD were used. Differences in gene content precluded alignment of the LPS biosynthetic gene cluster sequences across all genomes. Instead, the predicted amino acid sequences of the bordering *etfA* and *metB* genes were examined. For reference, a phylogenetic tree was generated from an alignment of the *rrnA* operon of each strain, rooted by including the *rrnA* operon of *Xylella fastidiosa* strain 9a–5c.

The *rrnA* tree groups the *Xanthomonas* strains into three distinct clades consisting of the *X. campestris* strains, the *X. oryzae* strains, and the *X. axonopodis* strains, in agreement with Rademaker et al. [14]. The *X. axonopodis* clade is basal, suggesting that these strains most closely resemble the common ancestor of the three clades. In the trees derived for each gene cluster, though the trees are not rooted and relative distances among sequences from different strains vary from those in the *rrnA* tree, the three clades are generally preserved. This shared overall topology indicates that the most recent common ancestor of the strains examined contained each of the clusters and that the current sequences are the result of evolution over the course of direct transmission. Exceptions to the shared topology are the positions of Xav and Xac in the *hrp* and *rpf* trees. In these trees, Xav lies between the *X. oryzae* clade and Xac, and Xac occupies a distinct, more distant branch. The nucleotide sequence of the core *hrp* genes (*hrcC* through *hpaB*) of Xav is more similar to that of Xoo (94% identity) than to that of Xac (92% identity). The Xac sequence is 99% identical to that of strain 8ra of *X. axonopodis* pv. glycines (GenBank accession AF499777). In the *rpf* tree, Xac is markedly distant from the other strains. Individual Rpf protein trees (not shown) indicate that this is due to highly distinct sequences for RpfF and RpfC in Xac. Also, the Xac cluster is missing *rpfH* and lacks any intergenic space between *rpfC* and *rpfG*. Thus, lateral acquisition and substitution of or within the *hrp* and *rpf* clusters may have taken place in the Xac or Xav lineages. It is also possible, though less likely based on the degree of divergence of the sequences, that the *X. axonopodis* clade, being the most basal in the phylogeny, acquired a greater degree of sequence diversity at these loci independent of lateral transfer.

Nevertheless, the exceptional sequence relationships for *hrp* and *rpf* genes in Xac and Xav do not correlate with host-or tissue-specificity within the group of strains examined. Also, with regard to host specificity, except for the *X. axonopodis* strains, which infect citrus and pepper, respectively, strains within clades infect the same or closely related hosts, so the topology where it is shared with that of the *rrnA* tree is not informative, except that there is no robust clustering of pathogens of monocots versus pathogens of eudicots. That is, there is no correlation of gene cluster sequence with the general class of host colonized, arguing against a defining role of any particular cluster in determining host specificity. With regard to tissue specificity, *X. campestris* and *X. oryzae* each contain vascular and non-vascular pathogens, yet across these species, none of the trees group pathogens that colonize the same tissues and therefore provide no evidence of a role in tissue specificity for any of the clusters. The similarity of the *etfA* and *metB* tree to the others indicates that the recombination that gave rise to the observed diversity of gene content at the LPS biosynthesis locus took place within or between these genes.

### Sequences predicted to be under selection

**Figure 3:**
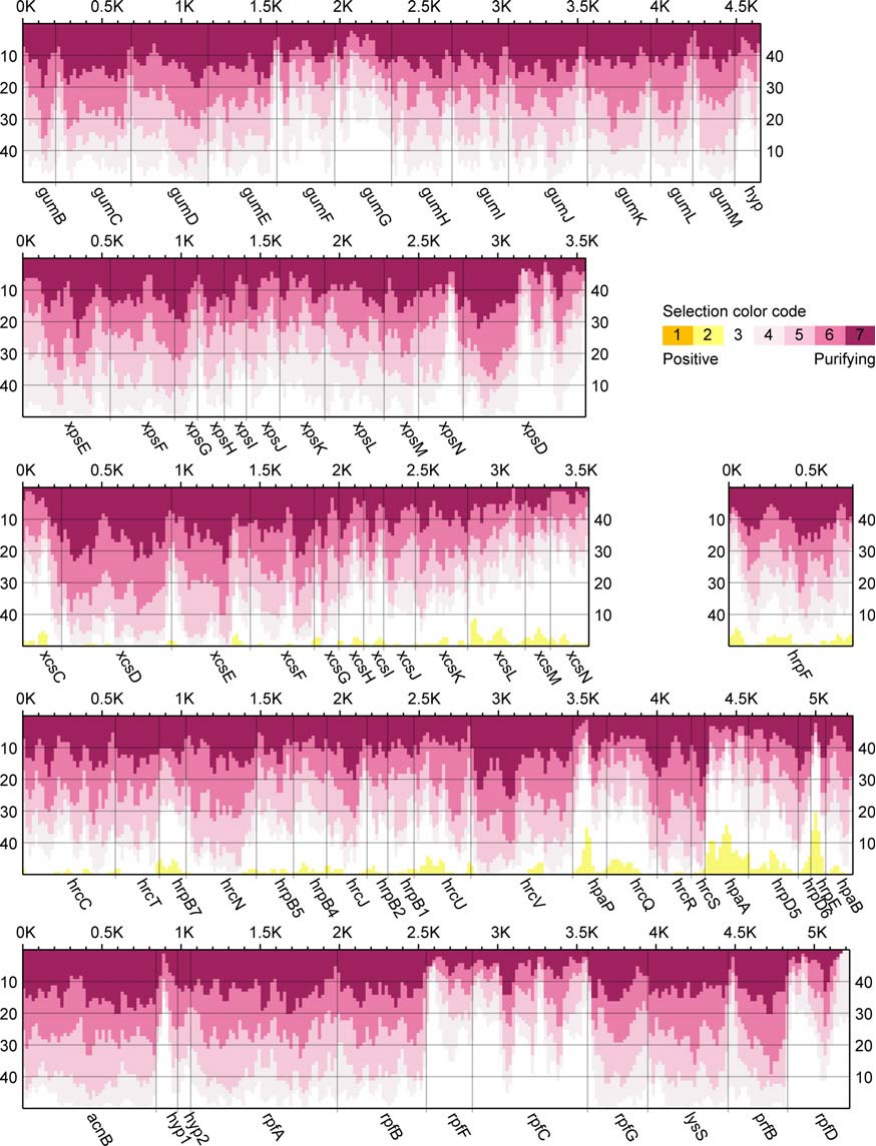
Sequences predicted to be under selection within the gene clusters examined. Based on the multiple alignments for each cluster, the Ka/Ks score for each codon was calculated with Selecton [101]. Shown is a plot of the Ka/Ks scores across each cluster using a window size of 50 with an offset of 20 residues, drawn using custom software. The vertical scales refer to the number of residues predicted to be under purifying (left) or positive (right) selection in each window. Evidence for selction (Ka/Ks ratios) is color coded, as shown at upper right, with yellow representing evidence of strong positive selection (high Ka/Ks ratio) and purple purifying selection (low Ka/Ks ratio). Raw selecton output for each alignment is available as Data S1.

To identify candidate sequences under selection during adaptation that led to the different *Xanthomonas* strains, we carried out an analysis of non-synonymous vs. synonymous substitutions in the multiple alignments of concatenated coding sequences in each cluster across genomes using the Selecton Web Server [101]. Most sequences showed evidence of purifying or no selection (Ka/Ks≤1), but codons in several genes in the *xcs* and *hrp* clusters showed evidence of positive selection, with the greatest concentration of such codons in *hpaP*, *hpaA*, and *hrpE* (**Figure 3**). High scores for residues in the *xcs* cluster alignments are considered tentative due to the small number of input sequences, which can result in artifact [101]. The *hpaP* gene (*hpaC* in Xav) encodes part of a bacterial intracellular protein complex that includes the global effector chaperone HpaB. This complex controls type III secretion of effector proteins and of non-effector translocon proteins, which function in translocation of effectors into the plant cell [66]. In Xav, HpaC distinguishes between two classes of effectors, only one of which is dependent on it for secretion. Divergence among HpaP/HpaC sequences could reflect different effector content across strains, which could be subject to positive selection via interactions with different plant hosts. The *hpaA* gene encodes a secreted and translocated protein that functions more broadly in the control of type III secretion, affecting the secretion of effectors and translocators, as well as the T3S pilus component HrpE [67], [102]. Binding of HpaA to HpaB is thought to block effector secretion and allow passage of non-effectors; secretion of HpaA is postulated then to liberate HpaB and initiate effector secretion [67]. In light of its effector non-discriminatory role in secretion, divergence among HpaA sequences likely relates to its plant intracellular function. Positive selection in *hrpE*, which encodes the T3S pilin subunit, was identified and discussed previously [103].

To address whether the evidence of positive selection we detected might relate to host- or tissue-specificity, we aligned sequences, both nucleotide and predicted amino acid, and constructed trees for each of the *xcs* and *hrp* genes individually. Except for HrcS and HrcN, none of the trees show relationships different from strain phylogeny, including the trees for *hpaP*, *hpaA*, and *hrpE*. The HrcS tree groups Xca more closely with the *X. axonopodis* strains than with the Xcc strains, suggestive of a correlation to tissue-specificity for those strains, but Xoc in that tree groups with the Xoo strains, and HrcS, as a predicted inner membrane, core component of the T3S apparatus, would not be expected to play a direct role in host interactions. The HrcN tree places the *X. oryzae* strains between Xav and Xac, but this relationship does not reflect host- or tissue-specificity, and HrcN, a cytoplasmic ATPase that drives T3S, like HrcS would not be expected to play a direct role in host interaction.

### Gene product polymorphisms correlated to tissue-specificity

Irrespective of evidence for positive selection, in the multiple alignment for each cluster, individual residues at each position were examined for polymorphism that correlated to tissue-specificity. To maximize the likelihood of detecting correlations, residues were scored for similarity using several different amino acid substitution matrices (see Materials and Methods). Across all alignments, four positions correlated with tissue-specificity, based on any matrix. These correspond to residue 131 in HpaA and residues 494, 696, and 698 in XpsD (**Table 2**; positions given relative to the Xoc sequences). The same analysis but with Xoc and Xoo switched in the groupings served as a control to assess the significance of the observed numbers of residues potentially involved in tissue-specificity. Because the control also indicated two positions (one in hrcU and one in XpsD), we cannot exclude the possibility that the residue differences correlated to tissue-specificity listed above are chance events. However, the identity of the genes in which the correlated positions are located, and the concentration of possible tissue-specificity determinants in the C-terminal domain of XpsD are intriguing.

**Table 2:** Amino acid residues in alignments of pathogenesis-associated gene products that correlate with tissue-specificity across eight *Xanthomonas* strains.

GENE	POS^a^	Vascular		Non-Vascular			
		Monocot	Dicot	Monocot	Dicot		
		Xoo^b^	Xcc	Xoc	Xca	Xac	Xav
*hpaA*	131	R	S	A	A	A	A
*xpsD*	494	K	R	Q	A	A	Q
*xpsD*	696	N	N	S	D	V	A
*xpsD*	698	I	I	V	V	V	L

a Position in the Xoc gene product.

b Strain abbreviations are as in the text. The Xoo and Xcc strains are vascular pathogens; Xoc, Xca, and the *X. axonopodis* strains are non-vascular pathogens.

As discussed earlier, HpaA is a substrate of the T3S system that also plays a role in controlling secretion of type III effector and translocator proteins, via interaction with HpaB. Residue 131 in HpaA (a 275 amino acid protein) is between the N-terminal secretion and translocation domain and the C-terminal HpaB-binding domain [67]. An effector function for HpaA has not yet been identified, but the abundance of positions showing evidence of positive selection and the correlation of residues at position 131 with tissue-specificity are consistent with an important, host-interactive role, and potential for residue 131 in particular, in determining the ability of the bacterium to colonize different host tissues.

XpsD is an outer membrane protein [104]. Members of the T2S:D protein family, to which XpsD belongs, are postulated to function as gatekeepers for type II secretion, demonstrating species-specific function for different type II secretion substrates [105], [106]. XpsD in different strains could direct the secretion of different sets of proteins adapted for function in different tissues, or, as a bacterial outer membrane protein, XpsD could function as an elicitor of tissue-specific plant responses that confer interaction specificity. Consistent with the latter hypothesis, two of the three positions in XpsD that correlate with tissue-specificity reside in the hypervariable C-terminal S domain. As demonstrated with PulD of *Klebsiella*, the S domain interacts with a specific lipoprotein (PulS in the case of PulD) that pilots it to the outer membrane and is thought to aide in homo-oligodimerization. In complex with this lipoprotein, the S domain is predicted to be largely exposed on the bacterial cell surface [107].

### Conclusion

Several pathogenesis-associated gene clusters across eight *Xanthomonas* strains were compared to assess potential contributions of these clusters to host- and tissue-specificity. The strains fall into three clades, corresponding to species, each containing two pathovars. Included in these pathovar pairs are pathogens that infect the same host with different tissue-specificity, as well as pathogens that infect different hosts, with shared tissue-specificity. One of the clades is made up of monocot pathogens, and the other two are pathogens of eudicots. One of the eudicot pathogen clades is more closely related to the monocot pathogen clade than to the other eudicot pathogen clade (**Figure 2**). For this broadly representative group of plant pathogenic *Xanthomonas* strains, adaptation to different plant hosts and specific tissues within a host does not include major alteration or exchange of content within any of the gene clusters examined. Complex relationships within an LPS biosynthesis gene cluster indicate a history of horizontal transfer events and diversifying selection, suggesting an adaptive role, but these relationships do not correlate with host- or tissue-specificity.

Positive selection is evident at sites in several genes in the *xcs* and *hrp* clusters. Nevertheless, none of the *xcs* or *hrp* genes individually, when compared across strains, showed relationships that group pathogens from different clades based on host specificity (i.e., eudicot vs. monocot pathogens) or tissue-specificity (i.e., vascular vs. non-vascular pathogens). Across all alignments, however, four positions showed correlation of amino acid residue identity with tissue specificity, revealing the T3S regulatory and putative effector gene *hpaA* and the type II secretory pathway gene *xpsD* as candidate tissue-specificity determinants.

**Figure 4:**
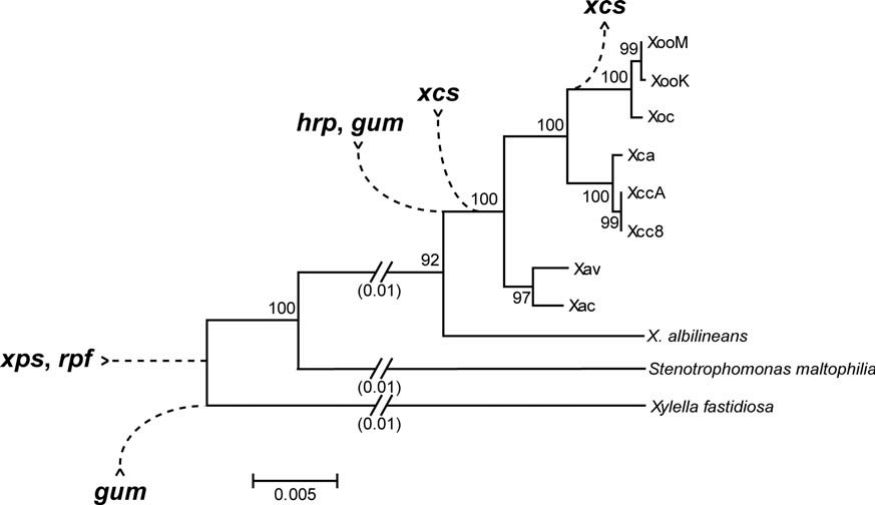
Inferred pattern of acquisition or loss of five pathogenesis-associated gene clusters in *Xanthomonas*. Based on comparison of genome sequences and other data among *Xanthomonas* strains and the close relatives *Xylella fastidiosa* and *Stenotrophomonas maltophilia* (see text), an inferred pattern of acquisition or loss of five pathogenesis associated gene clusters during the evolution of different *Xanthomonas* lineages is shown, superimposed on a phylogenetic tree drawn from an alignment of 16s rRNA gene sequences. Potential horizontal exchange of *hrp* and *rpf* sequences affecting the *X. axonopodis* clade, discussed in the text, is not depicted. Strain abbreviations are as in the text. For *X. fastidiosa*, the strain 9a–5c sequence was used. For *S. maltophilia*, the strain K279a sequence was used.

Comparison with other members of the Xanthomonadaceae revealed that the *rpf* and *xps* gene clusters were present early in the evolution of the group, that the *hrp*, *gum*, and *xcs* gene clusters were acquired later, and that the *xcs* cluster was subsequently lost in the lineage that gave rise to the *X. oryzae* clade (**Figure 4**). This pattern of acquisition and loss, coupled with the demonstrated importance of the *hrp* and *gum* clusters to pathogenesis in several *Xanthomonas* spp. and the lack of evidence for an important role of *xcs* genes in plant pathogen interactions, suggest that acquisition of the *hrp* and *gum* clusters were critical steps in the evolution of plant pathogenicity in *Xanthomonas*.

The results of our study provide insight into the nature of the first *Xanthomonas* genome, and suggest that differentiation of *Xanthomonas* species and pathovars with respect to host and tissue specificity resulted from subtle changes in a small number of individual genes in the *gum*, *hrp*, *xps*, *xcs*, or *rpf* clusters, modifications in non-coding, regulatory sequences in the clusters, and/or differences outside the clusters. Functional characterization of the differences discovered in *hpaA* and *xpsD*, expression analysis of the genes in each cluster, and examination of differences outside the clusters that correlate to host and tissue-specificity, particularly among regulatory targets or secretory substrates, or genes for environmental sensing, are important next steps.

## Methods

### Genome sequences and annotation

The genome sequences and annotation used are presented in **Table S1**. For some sequences, annotation was confirmed and refined manually by performing BLASTP comparisons against the non-redundant protein database (National Center for Biotechnology Information) and against other *Xanthomonas* genomes directly. Orthologs were defined as reciprocal best matches by BLASTP with an e-value minimum of e^−20^ and 60% coverage [19].

### Gene cluster comparisons

The genome clusters and corresponding genes were retrieved from genome sequences by referencing genome annotation. The coordinates for each cluster in each strain are presented in **Table S2**. Orthologous genes were grouped together, and in each group similarity to the gene in Xoc (if present) was calculated based on predicted amino acid sequence using the needle program of the EMBOSS package [108]. IS elements and tRNA genes were identified and mapped with BLASTN. Custom software was used to scan genome sequences to detect perfect and imperfect plant-inducible promoter sequences [PIP boxes, 109]. Overall sequence similarity of each cluster sequence to the Xoc sequence for that cluster was calculated using the stretcher program of the EMBOSS package [108]. GC content was plotted using a window size of 160 bp and a step size of 40 bp. In select comparisons, the genome of *Xylella fastidiosa* strain 9a–5c was included in the analysis but not shown. Schematic representations of the clusters were generated using custom software with the pre-calculated information above.

### Phylogenetic analyses

Concatenated protein sequences for each cluster and nucleotide sequences for the *rrnA* operon were aligned using ClustalW, Version 1.83, with default parameters [110]. Aligned sequences were inspected and manually adjusted when necessary. Regions with gaps between the strains were excluded to avoid problems reflecting start codon misassignment. Neighbor-joining trees were generated using PHYLIP [111] and displayed using Mega 3.1 [112]. Bootstrap values were derived from 1,000 replicates in each case to validate tree topology and are expressed as percent. Sequence from *Xylella fastidiosa* strain 9a–5c was used as an outgroup. For individual genes with codons showing evidence of positive selection, protein sequences were aligned using ClustalW, and trees were generated using PHYLIP. The PHYLIP programs PROTDIST and DNADIST, which use maximum likelihood estimates, were used to calculate distances, FITCH was used to estimate phylogenies from the distance matrices, and DRAWTREE was used to draw unrooted trees.

### Analysis of synonymous and non-synonymous substitutions

For analysis of synonymous and non-synonymous substitutions, nucleotide sequences of genes conserved across strains for each cluster were concatenated, using a 99 N spacer between individual gene sequences, and submitted to Selecton Version 2.4 (http://selecton.tau.ac.il) [101] with the Xoc sequence as consensus. The Mechanistic Empirical Combination (MEC) model was used with the “high precision” option selected. With custom software, Ka/Ks scores calculated by Selecton were plotted using a 50 amino acid window size and 20 amino acid offset. For each cluster that contained residues with evidence of positive selection (high Ka/Ks ratio), multiple alignments of each gene in the cluster across strains were generated and submitted to Selecton individually for confirmation, using the same parameters as above. For each of these multiple alignments, trees were also generated, as described above.

### Identification of gene product polymorphisms correlated to tissue-specificity

The multiple alignment of each cluster was scanned for positions at which residues in the Xcc and Xoo sequences vs. residues in the Xoc, Xca, Xac, and Xav sequences were more similar within these groups than across them. For this analysis, gaps in the alignments were retained. To minimize artifacts of alignment, only positions with at least one completely conserved neighbor were taken into consideration. The amino acid substitution matrices BLOSUM45, BLOSUM62, BLOSUM80, PAM30, PAM70 were used to assign substitution scores at each position in all pairwise comparisons, and then for each position the mean of the substitution scores within the groups of strains with like tissue-specificity, i.e., (Score(Xoo, Xcc)+Score(Xoc, Xca))/2, was compared with the mean of the scores for substitutions across groups of shared tissue specificity, i.e., (Score(Xoo, Xoc)+Score(Xoo, Xca)+Score(Xcc, Xoc)+Score(Xcc, Xca))/4. Any positions for which the mean within-group score was greater than the mean across-group score and at which there were no identical residues in any of the across-group comparisons were retained. For comparison to assess significance, these calculations were repeated using a tissue non-specific grouping of strains formed by switching Xoo with Xoc.

## Supporting Information

Supporting information includes 1) **Table S1**, *Xanthomonas* genome sequences examined in this study, 2) **Table S2**, Coordinates (bp) of the gene clusters examined in the eight *Xanthomonas* genomes, 3) **Text S1**, Additional details of gene cluster comparisons, and 4) **Data S1**, Raw Selecton output used to generate Figure 3.

Text S1 Additional details of gene cluster comparisons
(0.12 MB PDF)

Table S1 *Xanthomonas* genome sequences examined in this study.
(0.10 MB PDF)

Table S2 Coordinates (bp) of the gene clusters examined in the eight *Xanthomonas* genomes.
(0.06 MB PDF)

Data S1 Selecton results.
(0.10 MB ZIP)

## References

[1] Perna NT, Plunkett G, Burland V, Mau B, Glasner JD (2001). Genome sequence of enterohaemorrhagic *Escherichia coli* O157:H7.. Nature.

[2] Parkhill J, Wren BW, Mungall K, Ketley JM, Churcher C (2000). The genome sequence of the food-borne pathogen *Campylobacter jejuni* reveals hypervariable sequences.. Nature.

[3] Deng W, Burland V, Plunkett G, Boutin A, Mayhew GF (2002). Genome sequence of *Yersinia pestis* KIM.. J Bacteriol.

[4] Brosch R, Pym AS, Gordon SV, Cole ST (2001). The evolution of mycobacterial pathogenicity: clues from comparative genomics.. Trends Microbiol.

[5] Raskin DM, Seshadri R, Pukatzki SU, Mekalanos JJ (2006). Bacterial genomics and pathogen evolution.. Cell.

[6] Van Sluys MA, Monteiro-Vitorello CB, Camargo LEA, Menck CFM, da Silva ACR (2002). Comparative genomic analysis of plant-associated bacteria.. Annu Rev Phytopathol.

[7] Toth IK, Pritchard L, Birch PR (2006). Comparative genomics reveals what makes an enterobacterial plant pathogen.. Annu Rev Phytopathol.

[8] Comas I, Moya A, Azad RK, Lawrence JG, Gonzalez-Candelas F (2006). The evolutionary origin of Xanthomonadales genomes and the nature of the horizontal gene transfer process.. Mol Biol Evol.

[9] Lima WC, Sluys M-AV, Menck CFM (2005). Non-gamma-proteobacteria gene islands contribute to the *Xanthomonas* genome.. OMICS: J Integrative Biol.

[10] Lima WC, Paquola AC, Varani AM, Van Sluys MA, Menck CF (2008). Laterally transferred genomic islands in Xanthomonadales related to pathogenicity and primary metabolism.. FEMS Microbiol Lett.

[11] Hayward A, Swings J, Civerolo EL (1993). The hosts of *Xanthomonas*.. Xanthomonas.

[12] Goncalves ER, Rosato YB (2002). Phylogenetic analysis of *Xanthomonas* species based upon 16S-23S rDNA intergenic spacer sequences.. Int J Syst Evol Microbiol.

[13] Rademaker JLW, Hoste B, Louws FJ, Kersters K, Swings J (2000). Comparison of AFLP and rep-PCR genomic fingerprinting with DNA-DNA homology studies: *Xanthomonas* as a model system.. Int J Syst Evol Microbiol.

[14] Rademaker JLW, Louws FJ, Schultz MH, Rossbach U, Vauterin L (2005). A comprehensive species to strain taxonomic framework for *Xanthomonas*.. Phytopathology.

[15] Vauterin L, Hoste B, Kersters K, Swings J (1995). Reclassification of *Xanthomonas*.. Intl J Systematic Bacteriol.

[16] Nino-Liu DO, Ronald PC, Bogdanove AJ (2006). *Xanthomonas oryzae* pathovars: model pathogens of a model crop.. Mol Plant Pathol.

[17] Daniels MJ, Hopwood DA, Chater KF (1989). Pathogenicity of *Xanthomonas* and related bacteria towards plants.. Genetics of Bacterial Diversity.

[18] Qian W, Jia Y, Ren SX, He YQ, Feng JX (2005). Comparative and functional genomic analyses of the pathogenicity of phytopathogen *Xanthomonas campestris* pv. campestris.. Genome Res.

[19] da Silva AC, Ferro JA, Reinach FC, Farah CS, Furlan LR (2002). Comparison of the genomes of two *Xanthomonas* pathogens with differing host specificities.. Nature.

[20] Lee BM, Park YJ, Park DS, Kang HW, Kim JG (2005). The genome sequence of *Xanthomonas oryzae* pathovar oryzae KACC10331, the bacterial blight pathogen of rice.. Nucleic Acids Res.

[21] Ochiai H, Inoue Y, Takeya M, Sasaki A, Kaku H (2005). Genome sequence of *Xanthomonas oryzae* pv. oryzae suggests contribution of large numbers of effector genes and insertion sequences to its race diversity.. Jpn Agr Res Q.

[22] Thieme F, Koebnik R, Bekel T, Berger C, Boch J (2005). Insights into genome plasticity and pathogenicity of the plant pathogenic bacterium *Xanthomonas campestris* pv. vesicatoria revealed by the complete genome sequence.. J Bacteriol.

[23] Kamoun S, Kado CI (1990). Phenotypic switching affecting chemotaxis, xanthan production, and virulence in *Xanthomonas campestris*.. Appl Environ Microbiol.

[24] Raymundo AK, Leach JE (1993). Amplification and sequence analysis of the upstream region of the *hrpXo* gene homolog in *Xanthomonas oryzae* pv. oryzicola.. Philippine J Biotechnol.

[25] Salzberg SL, Sommer DD, Schatz MC, Phillippy AM, Rabinowicz PD (2008). Genome sequence and rapid evolution of the rice pathogen *Xanthomonas oryzae* pv. oryzae PXO99^A^.. BMC Genomics.

[26] Vorholter FJ, Schneiker S, Goesmann A, Krause L, Bekel T (2008). The genome of *Xanthomonas campestris* pv. campestris B100 and its use for the reconstruction of metabolic pathways involved in xanthan biosynthesis.. J Biotechnol.

[27] Gupta RS, Sneath PH (2007). Application of the character compatibility approach to generalized molecular sequence data: branching order of the proteobacterial subdivisions.. J Mol Evol.

[28] Bhattacharyya A, Stilwagen S, Ivanova N, D'Souza M, Bernal A (2002). Whole-genome comparative analysis of three phytopathogenic *Xylella fastidiosa* strains.. Proc Natl Acad Sci USA.

[29] Simpson AJ, Reinach FC, Arruda P, Abreu FA, Acencio M (2000). The genome sequence of the plant pathogen *Xylella fastidiosa*.. Nature.

[30] Van Sluys MA, de Oliveira MC, Monteiro-Vitorello CB, Miyaki CY, Furlan LR (2003). Comparative analyses of the complete genome sequences of Pierce's disease and citrus variegated chlorosis strains of *Xylella fastidiosa*.. J Bacteriol.

[31] Crossman LC, Gould VC, Dow JM, Vernikos GS, Okazaki A (2008). The complete genome, comparative and functional analysis of *Stenotrophomonas maltophilia* reveals an organism heavily shielded by drug resistance determinants.. Genome Biol.

[32] Purcell AH, Hopkins DL (1996). Fastidious xylem-limited bacterial plant pathogens.. Annu Rev Phytopathol.

[33] Berg G, Roskot N, Smalla K (1999). Genotypic and phenotypic relationships between clinical and environmental isolates of *Stenotrophomonas maltophilia*.. J Clin Microbiol.

[34] Vanderslice RW, Doherty DH, Capage MA, Betlach MR, Hassler RA, Crescenzi V, Dea ICM, Paoletti S, Stivala SS, Sutherland IW (1989). Genetic engineering of polysaccharide structure in *Xanthomonas campestris*.. Biomedical and Biotechnological Advances in Industrial Polysaccharides.

[35] Katzen F, Ferreiro DU, Oddo CG, Ielmini MV, Becker A (1998). *Xanthomonas campestris* pv. campestris *gum* mutants: effects on xanthan biosynthesis and plant virulence.. J Bacteriol.

[36] Dow JM, Daniels MJ, Dums F, Turner PC, Gough C (1989). Genetic and biochemical analysis of protein export from *Xanthomonas campestris*.. J Cell Sci Suppl.

[37] Arlat M, Gough CL, Barber CE, Boucher C, Daniels MJ (1991). *Xanthomonas campestris* contains a cluster of *hrp* genes related to the larger *hrp* cluster of *Pseudomonas solanacearum*.. Mol Plant-Microbe Interact.

[38] Dow JM, Crossman L, Findlay K, He YQ, Feng JX (2003). Biofilm dispersal in *Xanthomonas campestris* is controlled by cell-cell signaling and is required for full virulence to plants.. Proc Natl Acad Sci USA.

[39] Dharmapuri S, Yashitola J, Vishnupriya MR, Sonti RV (2001). Novel genomic locus with atypical G+C content that is required for extracellular polysaccharide production and virulence in *Xanthomonas oryzae* pv. oryzae.. Mol Plant-Microbe Interact.

[40] Vorholter FJ, Niehaus K, Puhler A (2001). Lipopolysaccharide biosynthesis in *Xanthomonas campestris* pv. campestris: a cluster of 15 genes is involved in the biosynthesis of the LPS O-antigen and the LPS core.. Mol Genet Genomics.

[41] Denny TP (1995). Involvement of bacterial polysaccharides in plant pathogenesis.. Annu Rev Phytopathol.

[42] Dharmapuri S, Sonti RV (1999). A transposon insertion in the *gumG* homologue of *Xanthomonas oryzae* pv. oryzae causes loss of extracellular polysaccharide production and virulence.. FEMS Microbiol Lett.

[43] Rigano LA, Siciliano F, Enrique R, Sendin L, Filippone P (2007). Biofilm formation, epiphytic fitness, and canker development in *Xanthomonas axonopodis* pv. citri.. Mol Plant-Microbe Interact.

[44] Dunger G, Relling VM, Tondo ML, Barreras M, Ielpi L (2007). Xanthan is not essential for pathogenicity in citrus canker but contributes to *Xanthomonas* epiphytic survival.. Arch Microbiol.

[45] Kiraly Z, El-Zahaby HM, Klement Z (1997). Role of extracellular polysaccharide (EPS) slime of plant pathogenic bacteria in protecting cells to reactive oxygen species.. J Phytopathol.

[46] Yoon KH, Cho JY (2007). Transcriptional analysis of the *gum* gene cluster from *Xanthomonas oryzae* pathovar oryzae.. Biotechnol Lett.

[47] Champoiseau P, Daugrois JH, Pieretti I, Cociancich S, Royer M (2006). High variation in pathogenicity of genetically closely related strains of *Xanthomonas albilineans*, the sugarcane leaf scald pathogen, in Guadeloupe.. Phytopathology.

[48] Fontaniella B, Rodriguez CW, Pinon D, Vicente C, Legaz M-E (2002). Identification of xanthans isolated from sugarcane juices obtained from scalded plants infected by *Xanthomonas albilineans*.. J Chromatogr B.

[49] da Silva FR, Vettore AL, Kemper EL, Leite A, Arruda P (2001). Fastidian gum: the *Xylella fastidiosa* exopolysaccharide possibly involved in bacterial pathogenicity.. FEMS Microbiol Lett.

[50] Blanch M, Legaz M-E, Vicente C (2008). Xanthan production by *Xanthomonas albilineans* infecting sugarcane stalks.. J Plant Physiol.

[51] d'Enfert C, Ryter A, Pugsley AP (1987). Cloning and expression in *Escherichia coli* of the *Klebsiella pneumoniae* genes for production, surface localization and secretion of the lipoprotein pullulanase.. EMBO J.

[52] Possot O, d'Enfert C, Reyss I, Pugsley AP (1992). Pullulanase secretion in *Escherichia coli* K-12 requires a cytoplasmic protein and a putative polytopic cytoplasmic membrane protein.. Mol Microbiol.

[53] Dums F, Dow JM, Daniels MJ (1991). Structural characterization of protein secretion genes of the bacterial phytopathogen *Xanthomonas campestris* pathovar campestris: relatedness to secretion systems of other gram-negative bacteria.. Mol Gen Genet.

[54] Ray SK, Rajeshwari R, Sharma Y, Sonti RV (2002). A high-molecular-weight outer membrane protein of *Xanthomonas oryzae* pv. oryzae exhibits similarity to non-fimbrial adhesins of animal pathogenic bacteria and is required for optimum virulence.. Mol Microbiol.

[55] Sun QH, Hu J, Huang GX, Ge C, Fang RX (2005). Type-II secretion pathway structural gene *xpsE*, xylanase- and cellulase secretion and virulence in *Xanthomonas oryzae* pv. oryzae.. Plant Pathol.

[56] Sandkvist M (2001). Type II secretion and pathogenesis.. Infect Immun.

[57] Brunings AM, Gabriel DW (2003). *Xanthomonas citri*: breaking the surface.. Mol Plant Pathol.

[58] Peabody CR, Chung YJ, Yen MR, Vidal-Ingigliardi D, Pugsley AP (2003). Type II protein secretion and its relationship to bacterial type IV pili and archaeal flagella.. Microbiology.

[59] Harding NE, Cleary JM, Cabanas DK, Rosen IG, Kang KS (1987). Genetic and physical analyses of a cluster of genes essential for xanthan gum biosynthesis in *Xanthomonas campestris*.. J Bacteriol.

[60] Moreira LM, De Souza RF, Almeida NF, Setubal JC, Oliveira JC (2004). Comparative genomics analyses of citrus-associated bacteria.. Annu Rev Phytopathol.

[61] Chen Y, Shiue SJ, Huang CW, Chang JL, Chien YL (2005). Structure and function of the XpsE N-terminal domain, an essential component of the *Xanthomonas campestris* type II secretion system.. J Biol Chem.

[62] Cornelis GR (2006). The type III secretion injectisome.. Nat Rev Microbiol.

[63] Sugio A, Yang B, White FF (2005). Characterization of the *hrpF* pathogenicity peninsula of *Xanthomonas oryzae* pv. oryzae.. Mol Plant-Microbe Interact.

[64] Bogdanove AJ, Beer SV, Bonas U, Boucher C, Collmer A (1996). Unified nomenclature for broadly conserved *hrp* genes of phytopathogenic bacteria.. Mol Microbiol.

[65] Kim JG, Park BK, Yoo CH, Jeon E, Oh J (2003). Characterization of the *Xanthomonas axonopodis* pv. glycines Hrp pathogenicity island.. J Bacteriol.

[66] Buttner D, Lorenz C, Weber E, Bonas U (2006). Targeting of two effector protein classes to the type III secretion system by a HpaC- and HpaB-dependent protein complex from *Xanthomonas campestris* pv. vesicatoria.. Mol Microbiol.

[67] Lorenz C, Kirchner O, Egler M, Stuttmann J, Bonas U (2008). HpaA from *Xanthomonas* is a regulator of type III secretion.. Mol Microbiol.

[68] Noel L, Thieme F, Nennstiel D, Bonas U (2002). Two novel type III-secreted proteins of *Xanthomonas campestris* pv. vesicatoria are encoded within the *hrp* pathogenicity island.. J Bacteriol.

[69] Leite RP, Minsavage GV, Bonas U, Stall RE (1994). Detection and identification of phytopathogenic *Xanthomonas* strains by amplification of DNA sequences related to the *hrp* genes of *Xanthomonas campestris* pv. vesicatoria.. Appl Environ Microbiol.

[70] Moore ER, Kruger AS, Hauben L, Seal SE, Daniels MJ (1997). 16S rRNA gene sequence analyses and inter- and intrageneric relationships of *Xanthomonas* species and *Stenotrophomonas maltophilia*.. FEMS Microbiol Lett.

[71] Birch RG, Patil SS (1987). Correlation between albicidin production and chlorosis induction by *Xanthomonas albilineans*, the sugarcane leaf scald pathogen.. Physiol Mol Plant Pathol.

[72] Birch RG, Patil SS (1987). Evidence that an albicidin-like phytotoxin induces chlorosis in sugarcane leaf scald disease by blocking plastid DNA replication.. Physiol Mol Plant Pathol.

[73] Dow JM, Daniels MJ (2000). *Xylella* genomics and bacterial pathogenicity to plants.. Yeast.

[74] Barber CE, Tang JL, Feng JX, Pan MQ, Wilson TJ (1997). A novel regulatory system required for pathogenicity of *Xanthomonas campestris* is mediated by a small diffusible signal molecule.. Mol Microbiol.

[75] Dow JM, Feng JX, Barber CE, Tang JL, Daniels MJ (2000). Novel genes involved in the regulation of pathogenicity factor production within the *rpf* gene cluster of *Xanthomonas campestris*.. Microbiology.

[76] Slater H, Alvarez-Morales A, Barber CE, Daniels MJ, Dow JM (2000). A two-component system involving an HD-GYP domain protein links cell-cell signalling to pathogenicity gene expression in *Xanthomonas campestris*.. Mol Microbiol.

[77] Wilson TJG, Bertrand N, Tang JL, Feng JX, Pan MQ (1998). The *rpfA* gene of *Xanthomonas campestris* pathovar campestris, which is involved in the regulation of pathogenicity factor production, encodes an aconitase.. Mol Microbiol.

[78] Wang LH, He YW, Gao YF, Wu JE, Dong YH (2004). A bacterial cell-cell communication signal with cross-kingdom structural analogues.. Mol Microbiol.

[79] Ryan RP, Fouhy Y, Lucey JF, Crossman LC, Spiro S (2006). Cell-cell signaling in *Xanthomonas campestris* involves an HD-GYP domain protein that functions in cyclic di-GMP turnover.. Proc Natl Acad Sci USA.

[80] Chatterjee S, Sonti RV (2002). *rpfF* mutants of *Xanthomonas oryzae* pv. oryzae are deficient for virulence and growth under low iron conditions.. Mol Plant-Microbe Interact.

[81] Siciliano F, Torres P, Sendín L, Bermejo C, Filippone P (2006). Analysis of the molecular basis of *Xanthomonas axonopodis* pv. *citri* pathogenesis in *Citrus limon*.. Electron J Biotechnol.

[82] Tang JL, Feng JX, Li QQ, Wen HX, Zhou DL (1996). Cloning and characterization of the *rpfC* gene of *Xanthomonas oryzae* pv oryzae: involvement in exopolysaccharide production and virulence to rice.. Mol Plant-Microbe Interact.

[83] Wang L, Makino S, Subedee A, Bogdanove AJ (2007). Novel candidate virulence factors in rice pathogen *Xanthomonas oryzae* pv. *oryzicola* as revealed by mutational analysis.. Appl Environ Microbiol.

[84] Boon C, Deng Y, Wang LH, He Y, Xu JL (2008). A novel DSF-like signal from *Burkholderia cenocepacia* interferes with *Candida albicans* morphological transition.. ISME J.

[85] Scarpari LM, Lambais MR, Silva DS, Carraro DM, Carrer H (2003). Expression of putative pathogenicity-related genes in *Xylella fastidiosa* grown at low and high cell density conditions *in vitro*.. FEMS Microbiol Lett.

[86] Simionato AVC, da Silva DS, Lambais MR, Carrilho E (2007). Characterization of a putative *Xylella fastidiosa* diffusible signal factor by HRGC-EI-MS.. J Mass Spectrom.

[87] Fouhy Y, Scanlon K, Schouest K, Spillane C, Crossman L (2007). Diffusible signal factor-dependent cell-cell signaling and virulence in the nosocomial pathogen *Stenotrophomonas maltophilia*.. J Bacteriol.

[88] Raetz CR, Whitfield C (2002). Lipopolysaccharide endotoxins.. Annu Rev Biochem.

[89] Mooi FR, Bik EM (1997). The evolution of epidemic *Vibrio cholerae* strains.. Trends Microbiol.

[90] Reeves PP, Wang L (2002). Genomic organization of LPS-specific loci.. Curr Top Microbiol Immunol.

[91] Newman MA, Dow JM, Daniels MJ (2001). Bacterial lipopolysaccharides and plant-pathogen interactions.. Eur J Plant Pathol.

[92] Medzhitov R, Janeway CA (1997). Innate Immunity: The virtues of a nonclonal system of recognition.. Cell.

[93] Mackey D, McFall AJ (2006). MAMPs and MIMPs: proposed classifications for inducers of innate immunity.. Mol Microbiol.

[94] Silipo A, Molinaro A, Sturiale L, Dow JM, Erbs G (2005). The elicitation of plant innate immunity by lipooligosaccharide of *Xanthomonas campestris*.. J Biol Chem.

[95] Bedini E, De Castro C, Erbs G, Mangoni L, Dow JM (2005). Structure-dependent modulation of a pathogen response in plants by synthetic O-antigen polysaccharides.. J Am Chem Soc.

[96] Lerouge I, Vanderleyden J (2002). O-antigen structural variation: mechanisms and possible roles in animal/plant-microbe interactions.. FEMS Microbiol Rev.

[97] Patil PB, Sonti RV (2004). Variation suggestive of horizontal gene transfer at a lipopolysaccharide (*lps*) biosynthetic locus in *Xanthomonas oryzae* pv. oryzae, the bacterial leaf blight pathogen of rice.. BMC Microbiol.

[98] Koplin R, Arnold W, Hotte B, Simon R, Wang G (1992). Genetics of xanthan production in *Xanthomonas campestris*: the *xanA* and *xanB* genes are involved in UDP-glucose and GDP-mannose biosynthesis.. J Bacteriol.

[99] Steinmann D, Koplin R, Puhler A, Niehaus K (1997). *Xanthomonas campestris* pv. campestris *lpsI* and *lpsJ* genes encoding putative proteins with sequence similarity to the alpha- and beta-subunits of 3-oxoacid CoA-transferases are involved in LPS biosynthesis.. Arch Microbiol.

[100] Patil PB, Bogdanove AJ, Sonti RV (2007). The role of horizontal transfer in the evolution of a highly variable lipopolysaccharide biosynthesis locus in xanthomonads that infect rice, citrus and crucifers.. BMC Evol Biol.

[101] Stern A, Doron-Faigenboim A, Erez E, Martz E, Bacharach E (2007). Selecton 2007: advanced models for detecting positive and purifying selection using a Bayesian inference approach.. Nucleic Acids Res.

[102] Huguet E, Hahn K, Wengelnik K, Bonas U (1998). *hpaA* mutants of *Xanthomonas campestris* pv. vesicatoria are affected in pathogenicity but retain the ability to induce host-specific hypersensitive reaction.. Mol Microbiol.

[103] Weber E, Koebnik R (2005). Domain structure of HrpE, the Hrp pilus subunit of *Xanthomonas campestris* pv. vesicatoria.. J Bacteriol.

[104] Chen LY, Chen DY, Miaw J, Hu NT (1996). XpsD, an outer membrane protein required for protein secretion by *Xanthomonas campestris* pv. campestris, forms a multimer.. J Biol Chem.

[105] Lindeberg M, Salmond GP, Collmer A (1996). Complementation of deletion mutations in a cloned functional cluster of *Erwinia chrysanthemi out* genes with *Erwinia carotovora out* homologues reveals OutC and OutD as candidate gatekeepers of species-specific secretion of proteins via the type II pathway.. Mol Microbiol.

[106] Bouley J, Condemine G, Shevchik VE (2001). The PDZ domain of OutC and the N-terminal region of OutD determine the secretion specificity of the type II out pathway of *Erwinia chrysanthemi*.. J Mol Biol.

[107] Nouwen N, Ranson N, Saibil H, Wolpensinger B, Engel A (1999). Secretin PulD: association with pilot PulS, structure, and ion-conducting channel formation.. Proc Natl Acad Sci U S A.

[108] Rice P, Longden I, Bleasby A (2000). EMBOSS: the European Molecular Biology Open Software Suite.. Trends Genet.

[109] Tsuge S, Terashima S, Furutani A, Ochiai H, Oku T (2005). Effects on promoter activity of base substitutions in the cis-acting regulatory element of *HrpXo* regulons in *Xanthomonas oryzae* pv. oryzae.. J Bacteriol.

[110] Thompson JD, Higgins DG, Gibson TJ (1994). CLUSTAL W: improving the sensitivity of progressive multiple sequence alignment through sequence weighting, position-specific gap penalties and weight matrix choice.. Nucleic Acids Res.

[111] Felsenstein J (2007). PHYLIP (Phylogeny Inference Package) version 3.67. Distributed by the author..

[112] Kumar S, Tamura K, Nei M (2004). MEGA3: Integrated software for Molecular Evolutionary Genetics Analysis and sequence alignment.. Brief Bioinform.

