# Supplementary material for: Acquisition and Evolution of Plant Pathogenesis–Associated Gene Clusters and Candidate Determinants of Tissue-Specificity in Xanthomonas
Source: PLoS One. 2008 Nov 27;3(11):e3828. doi: 10.1371/journal.pone.0003828 (PMC2585010; doi:10.1371/journal.pone.0003828)
Supplement: Text S1 — Additional details of gene cluster comparisons (0.12 MB PDF) [file pone.0003828.s001.pdf]

Text S1 - Additional details of gene cluster comparisons

The *gum* gene cluster

In Xav, *gumN* is disrupted by an insertion sequence (IS) element, *IS476*. In XooK and XooM there are two IS elements, *ISXo7* and *ISXoo16*, downstream of *gumN*, which is intact. These IS elements are not present, however, in Xoc. Instead, in Xoc, *gumN* is broken into two likely non-functional ORFs separated by apparently vestigial sequences of *IS476*, suggestive of insertion and subsequent imperfect excision. Downstream of *gumN* in Xac, Xcc, and Xca is a short non-coding region followed by *gumO*, *gumP* and a third gene, *cdh2* (encoding NAD(P)H steroid dehydrogenase) in an arrangement that suggests co-transcription. In Xav, *cdh2* is missing and not found elsewhere in the genome. In Xoc, *cdh2* and *gumP* are missing, and *gumO* is truncated. In XooK and XooM, all three genes are absent. At the other end of the cluster, between *gumB* and *gumA,* a tRNA-Pro is present in all eight strains.

The most divergent intact gene in the cluster is *gumG*: percent amino acid identity across strains ranges from 66.0 to 96.4. This gene encodes an acyltransferase proposed to target the external mannose residue of a lipid-linked pentasaccharide xanthan intermediate [1]. In each cluster, GumG shows roughly 40% identity with GumF. *gumG* is not present in the *gum* gene cluster of *Xylella fastidiosa*. Thus, this gene is almost certainly a duplication of *gumF*, which could explain its greater divergence among strains: if functionally redundant to *gumF*, *gumG* would not be under selective constraints and could accumulate more mutations. Conceivably, differences in the activity of different GumG orthologs could result in differential acylation of xanthan in different strains and affect interactions with plants. Nonetheless, ablation of *gumG* caused only a moderate reduction in virulence in Xcc [1], and relationships among the GumG orthologs from the strains examined do not differ from the overall phylogeny (see below), suggesting that *gumG* does not play a role in determining interaction specificity.

A role for *gumO* and *gumP* in host-specificity related to EPS structural variation is conceivable based on the unique presence of these genes in the pathogens of eudicots. However, no mutations associated with virulence that map to these genes have been reported, and their predicted functions based on BLAST matches to characterized protein families (TIGR00747, 3-oxoacyl-(acyl-carrier-protein) synthase III, and PF00753, metallo-beta-lactamase, respectively) do not appear to relate to xanthan biosynthesis.

The *hrp* gene cluster

The majority of *hrp* and *hrc* genes are present in Region I, extending from *hpa2* to *hpaB*, in the same order and orientation across strains, suggesting that this represents the ancestral arrangement. An argument can be made for the ancestral cluster also containing the gene immediately downstream of *hpa2,* designated here as *hpa5*, because related sequences can be found in all of the genomes, and because *hpa5* is preceded by a candidate PIP (plant inducible promoter) box, a conserved regulatory element found upstream of *hpa1, hrpD5, hrcQ, hrcU* and *hrpB1*, and in degenerate form upstream of *hpa2* [2]. Interestingly, the integrity of *hpa5* as represented in Xoc was not maintained in most of the genomes. For example, the *X. campestris* strains share an apparent multiple gene insertion event within the *hpa5* sequence. Two of the inserted ORFs (XCC1246 and XCC1247 in XccA) share sequence relatedness with *eop3* and *xopP*, respectively, of *Erwinia amylovora* and Xav, and therefore may encode type III-secreted

effector proteins [3,4]. Xav is exceptional in Region I in that a type III effector gene named *xopD*, flanked by repetitive elements, is inserted between *hrcC* and *hpa1*.

Region II is centered on *hrpF*, conserved in all genomes and the only known critical gene of the region. Most genomes also contain *xopF1* or remnants and an associated ORF referred to here as *hpa3*. When present, *hpa3* is preceded by a near-perfect PIP box and may be co-transcribed with *xopF1*, which has features of a type III effector protein in Xav [4]. *xopF1* and *hpa3* may represent the ancestral Region II since they are present in Xoc, XooM, Xav, and Xca, which represent each of the three *Xanthomonas* species examined. The Xcc strains are missing *hpa3* and the N-terminal coding region of *xopF1*. Xac has an apparent frameshift mutation in *xopF1*. In keeping with their close ancestry, however, Xca and the Xcc strains share some differences from the other strains in Region II. They lack *hpaF* loci, and *hrpF* is oriented opposite in relation to the other strains. Additionally, *hrpW*, either not present (Xav, Xoc, XooK, and XooM) or unlinked (Xac) to the *hrp* gene cluster in other strains, is present at the Region I–proximal border of Region II in the *X. campestris* strains. Region II in Xca contains an additional, large ORF, downstream of *hrpF*, which is related to candidate type III effector genes of the SKWP family [5]. In Xav, the full-length *hpaF* is represented by two genes, *hpaF* and *hpaG*, suggesting that this strain suffered a mutation that split the ORF into two.

The *rpf* gene cluster

The *rpfF* and *rpfC* genes in Xac are somewhat divergent relative to their counterparts in the other genomes. Variations in the remainder of the *rpf* gene cluster are more pronounced (**Figure 1D**). *rpfH* is intact only in Xav and the *X. campestris* strains. The gene is completely missing from Xac and from the *rpf* cluster of the Xf and Sma genomes, but remnants are present in the *X. oryzae* strains. Though not conclusive, these observations suggest that *rpfH* is ancestral but has been lost independently in different lineages. The *rpfD* gene, though present in each genome, displays the most sequence variation, suggesting a possible adaptive role, but relationships within the gene family (not shown) mirror those based on rDNA alignments (see below). Immediately downstream of *rpfD* in Xoc resides a membrane protein–encoding gene that is truncated relative to its orthologs, which are present in all of the other strains. In the *X. campestris* strains, *rpfD* and this gene are separated by two short ORFs also predicted to encode membrane proteins. Downstream, the *rpfI* gene is present in Xoc and the *X. campestris* strains but absent from Xav and Xac. In XooK and XooM *rpfI* carries three internal frame shifts and is adjacent to a small region with IS elements. This region in Xac contains IS elements and a homolog of the *wapA* gene required for biofilm formation and attachment in the dental pathogen *Streptococcus mutans* [6].

References

1. Katzen F, Ferreiro DU, Oddo CG, Ielmini MV, Becker A, et al. (1998) *Xanthomonas campestris* pv. campestris *gum* mutants: effects on xanthan biosynthesis and plant virulence. J Bacteriol 180: 1607-1617.
2. Fenselau S, Bonas U (1995) Sequence and expression analysis of the *hrpB* pathogenicity operon of *Xanthomonas campestris* pv. vesicatoria which encodes eight proteins with similarity to components of the Hrp, Ysc, Spa, and Fli secretion systems. Mol Plant-Microbe Interact 8: 845-854.

3. Nissinen RM, Ytterberg AJ, Bogdanove AJ, Van Wijk KJ, Beer SV (2007) Analyses of the secretomes of *Erwinia amylovora* and selected *hrp* mutants reveal novel type III secreted proteins and an effect of HrpJ on extracellular harpin levels. Mol Plant Pathol 8: 55-67.
4. Roden JA, Belt B, Ross JB, Tachibana T, Vargas J, et al. (2004) A genetic screen to isolate type III effectors translocated into pepper cells during *Xanthomonas* infection. Proc Natl Acad Sci USA 101: 16624-16629.
5. Guidot A, Prior P, Schoenfeld J, Carrere S, Genin S, et al. (2007) Genomic structure and phylogeny of the plant pathogen *Ralstonia solanacearum* inferred from gene distribution analysis. J Bacteriol 189: 377-387.
6. Zhu L, Kreth J, Cross SE, Gimzewski JK, Shi W, et al. (2006) Functional characterization of cell-wall-associated protein WapA in *Streptococcus mutans*. Microbiology 152: 2395-2404.
