# Supplementary material for: Acquisition and Evolution of Plant Pathogenesis–Associated Gene Clusters and Candidate Determinants of Tissue-Specificity in Xanthomonas
Source: PLoS One. 2008 Nov 27;3(11):e3828. doi: 10.1371/journal.pone.0003828 (PMC2585010; doi:10.1371/journal.pone.0003828)
Supplement: Table S1 — Xanthomonas genome sequences examined in this study. (0.10 MB PDF) [file pone.0003828.s002.pdf]

Table S1. *Xanthomonas* genome sequences examined in this study[a].

Organism	Disease	Abbr.	Size (Mb)	Components	% G+C	% coding	Genes	GenBank Accession(s)	Ref.
Xanthomonas axonopodis pv. *citri* 306	Citrus canker	Xac	5.27	Circular chromosome (5,175,554 bp), plasmids pXAC64 (64,920 bp), pXAC33 (33,700 bp)	64.8	90.3	5,809	NC_003919, NC_003922, NC_003921	[1]
X. axonopodis pv. *vesicatoria (X. campestris* pv. *vesicatoria)* 85-10	Bacterial spot disease of pepper and tomato	Xav	5.42	Circular chromosome (5,178,466 bp), plasmids pXCV183 (182,572 bp), pXCV38 (38,116 bp), pXCV19 (19,146 bp), pXCV2 (1,852 bp)	64.6	86.6	5,229	NC_007508, NC_007507, NC_007506, NC_007505, NC_007504	[2]
X. campestris pv. *campestris* 8004	Black rot of crucifers	Xcc8	5.15	Circular chromosome	65.0	87.2	5,079	NC_007086	[3]
X. campestris pv. *campestris* ATCC33913	Black rot of crucifers	XccA	5.08	Circular chromosome	65.1	90.1	5,832	NC_003902	[1]
X. campestris pv. *armoraciae* 756C	Leaf spot disease of crucifers	Xca	4.94	Circular chromosome	65.3	85.3	4,598	Pending[b]	[4]
X. oryzae pv. *oryzae* KACC10331	Bacterial blight of rice	XooK	4.94	Circular chromosome	63.7	87.6	5,805	NC_006834	[5]
X. oryzae pv. *oryzae* MAFF311018	Bacterial blight of rice	XooM	4.94	Circular chromosome	63.7	83.9	5,091	NC_007705	[6]

Organism	Disease	Abbr.	Size (Mb)	Components	% G+C	% coding	Genes	GenBank Accession(s)	Ref.
X. oryzae pv. *oryzicola* BLS256	Bacterial leaf streak of rice	Xoc	4.83	Circular chromosome	64.0	86.0	4,686	AAQN01000001[c]	[4]

[a] Genome statistics were derived from the TIGR automated annotation for each genome available through the Comprehensive Microbial Resource (CMR; http://cmr.jcvi.org).
[b] Finished sequence and draft annotation are available through the CMR.
[c] Finished sequence only. Finished sequence and draft annotation are available through the CMR.

References

1. da Silva AC, Ferro JA, Reinach FC, Farah CS, Furlan LR, et al. (2002) Comparison of the genomes of two *Xanthomonas* pathogens with differing host specificities. Nature 417: 459-463.
2. Thieme F, Koebnik R, Bekel T, Berger C, Boch J, et al. (2005) Insights into genome plasticity and pathogenicity of the plant pathogenic bacterium *Xanthomonas campestris* pv. vesicatoria revealed by the complete genome sequence. J Bacteriol 187: 7254-7266.
3. Qian W, Jia Y, Ren SX, He YQ, Feng JX, et al. (2005) Comparative and functional genomic analyses of the pathogenicity of phytopathogen *Xanthomonas campestris* pv. campestris. Genome Res 15: 757-767.
4. AB et al, unpublished.
5. Lee BM, Park YJ, Park DS, Kang HW, Kim JG, et al. (2005) The genome sequence of *Xanthomonas oryzae* pathovar oryzae KACC10331, the bacterial blight pathogen of rice. Nucleic Acids Res 33: 577-586.
6. Ochiai H, Inoue Y, Takeya M, Sasaki A, Kaku H (2005) Genome sequence of *Xanthomonas oryzae* pv. oryzae suggests contribution of large numbers of effector genes and insertion sequences to its race diversity. Jpn Agr Res Q 39: 275-287.
