# Supplementary material for: Acquisition and Evolution of Plant Pathogenesis–Associated Gene Clusters and Candidate Determinants of Tissue-Specificity in Xanthomonas
Source: PLoS One. 2008 Nov 27;3(11):e3828. doi: 10.1371/journal.pone.0003828 (PMC2585010; doi:10.1371/journal.pone.0003828)
Supplement: Table S2 — Coordinates (bp) of the gene clusters examined in the eight Xanthomonas genomes. (0.06 MB PDF) [file pone.0003828.s003.pdf]

Table S2. Coordinates (bp) of the gene clusters examined in the eight *Xanthomonas* genomes.

Genome[a]	*gum*	*xps*	*xcs*	*hrp*	*rpf*	LPS
Xac	3,028192-3,048,738	4,178,455-4,189,560	813,305-845,755	462,612-491,050	2,160,878-2,191,415	4,253,203-4,274,866
Xav	3,148,901-3,169,454	4,213,203-4,224,466	852,276-874,639	459,855-495,309	2,158,277-2,180,861	4,286,730-4,307,761
Xca	2,900,826-2,921,324	846,986-861,263	4,013,878-4,033,581	1,481,968-1,529,914	2,158,303-2,183,043	774,891-794,777
Xcc8	1,992,417-2,012,926	4,230,259-4,241,536	877,383-897,083	3,588,956-3,622,061	2,810,926-2,835,681	4,296,386-4,318,442
XccA	2,898,293-2,918,802	799,012-810,289	4,067,649-4,087,364	1,421,715-1,454,821	2,147,236-2,171,985	719,235-742,650
Xoc	1,841,346-1,861,882	3,917,322-3,928,637	746,437-761,088	4,562,703-4,594,229	2,246,801-2,269,816	3,982,619-4,009,081
XooK	3,397,334-3,417,874	868,233-879,488	4,182,036-4,198,870	58,173-95,124	3,070,712-3,098,733	792,353-806,716
XooM	3,403,842-3,424,382	837,539-848,845	4,178,615-4,195,434	81,836-118,742	3,063,489-3,091,473	760,713-775,066

[a] Strain abbreviations are as in the text.
