# Supplementary material for: Acquisition and Evolution of Plant Pathogenesis–Associated Gene Clusters and Candidate Determinants of Tissue-Specificity in Xanthomonas
Source: PLoS One. 2008 Nov 27;3(11):e3828. doi: 10.1371/journal.pone.0003828 (PMC2585010; doi:10.1371/journal.pone.0003828)
Supplement: Data S1 — Selecton results. (0.10 MB ZIP) [file pone.0003828.s004.zip › Supplementary data - Selecton results/hrpF_ver04.html]

Selecton Results: 1207254893

# Selecton Results

  
  

|
|  |
| 1 | 11 | 21 | 31 | 41 |
| **M** **S** **L** **N** **M** **L** **S** **T** **G** **S** | **N** **P** **S** **Q** **L** **L** **G** **T** **S** **S** | **N** **E** **S** **S** **S** **S** **D** **L** **F** **G** | **S** **D** **S** **S** **R** **D** **G** **F** **D** **L** | **P** **S** **T** **M** **D** **T** **L** **F** **Q** **Q** |
|
| 51 | 61 | 71 | 81 | 91 |
| **I** **Y** **L** **V** **L** **A** **A** **L** **Q** **A** | **N** **T** **Q** **T** **G** **A** **S** **G** **D** **T** | **P** **A** **N** **T** **A** **S** **G** **D** **A** **D** | **T** **Q** **M** **S** **A** **S** **D** **W** **Q** **A** | **T** **Q** **P** **I** **E** **K** **R** **T** **S** **W** |
|
| 101 | 111 | 121 | 131 | 141 |
| **P** **S** **L** **D** **Y** **D** **F** **D** **P** **K** | **N** **I** **K** **G** **K** **D** **A** **P** **P** **A** | **L** **E** **G** **S** **T** **V** **T** **W** **N** **D** | **G** **T** **L** **T** **K** **S** **E** **L** **Q** **I** | **V** **S** **T** **L** **N** **A** **H** **K** **D** **Q** |
|
| 151 | 161 | 171 | 181 | 191 |
| **M** **P** **L** **E** **Y** **K** **N** **L** **D** **D** | **K** **I** **N** **D** **P** **S** **T** **P** **P** **D** | **L** **K** **A** **A** **L** **Q** **G** **L** **K** **Q** | **D** **P** **R** **L** **F** **F** **A** **I** **G** **S** | **Q** **G** **D** **G** **K** **C** **G** **G** **K** **V** |
|
| 201 | 211 | 221 | 231 | 241 |
| **S** **A** **Q** **D** **L** **W** **D** **F** **S** **D** | **S** **H** **P** **Q** **V** **K** **D** **L** **G** **G** | **K** **N** **D** **E** **F** **N** **P** **K** **D** **I** | **K** **G** **S** **N** **P** **P** **Q** **A** **A** **E** | **G** **S** **T** **V** **T** **W** **N** **D** **G** **Q** |
|
| 251 | 261 | 271 | 281 | 291 |
| **L** **N** **Q** **S** **E** **L** **E** **I** **V** **S** | **V** **L** **D** **R** **H** **K** **D** **Q** **V** **D** | **S** **L** **S** **F** **D** **Q** **L** **D** **A** **K** | **I** **N** **D** **P** **S** **T** **Q** **P** **D** **L** | **K** **E** **A** **L** **K** **G** **L** **Q** **K** **D** |
|
| 301 | 311 | 321 | 331 | 341 |
| **P** **R** **L** **F** **F** **A** **I** **G** **S** **Q** | **K** **D** **G** **K** **C** **G** **G** **K** **I** **K** | **A** **Q** **D** **L** **T** **D** **F** **S** **Y** **Y** | **H** **P** **Q** **I** **A** **E** **Y** **N** **D** **K** | **K** **A** **K** **S** **Y** **T** **Q** **N** **Y** **I** |
|
| 351 | 361 | 371 | 381 | 391 |
| **A** **S** **D** **S** **P** **D** **K** **T** **K** **A** | **S** **V** **M** **T** **K** **S** **D** **A** **L** **R** | **E** **M** **Y** **R** **Y** **S** **D** **Y** **L** **P** | **G** **N** **L** **S** **E** **D** **E** **F** **A** **K** | **I** **V** **D** **G** **D** **S** **K** **T** **G** **K** |
|
| 401 | 411 | 421 | 431 | 441 |
| **C** **P** **P** **Q** **L** **I** **A** **A** **A** **Q** | **Y** **F** **R** **D** **H** **P** **D** **E** **W** **K** | **E** **F** **S** **G** **D** **A** **G** **T** **M** **S** | **T** **P** **D** **F** **L** **Q** **K** **S** **T** **S** | **E** **M** **H** **L** **T** **A** **D** **E** **Q** **K** |
|
| 451 | 461 | 471 | 481 | 491 |
| **T** **L** **D** **T** **I** **N** **S** **H** **Q** **D** | **A** **F** **Y** **G** **D** **G** **K** **E** **L** **T** | **R** **D** **K** **L** **D** **A** **I** **S** **K** **D** | **D** **K** **A** **D** **P** **A** **V** **K** **E** **A** | **A** **T** **Q** **L** **A** **S** **D** **P** **L** **L** |
|
| 501 | 511 | 521 | 531 | 541 |
| **F** **G** **L** **L** **N** **N** **S** **I** **T** **G** | **Y** **K** **K** **P** **H** **H** **F** **F** **G** **G** | **G** **H** **V** **V** **D** **S** **G** **K** **I** **S** | **Q** **N** **D** **F** **R** **Q** **F** **Y** **D** **H** | **M** **S** **A** **A** **N** **K** **T** **V** **N** **T** |
|
| 551 | 561 | 571 | 581 | 591 |
| **P** **A** **T** **H** **E** **A** **S** **S** **P** **D** | **Q** **Q** **K** **A** **V** **A** **D** **M** **L** **M** | **G** **K** **D** **D** **P** **P** **A** **I** **K** **K** | **P** **K** **K** **D** **V** **G** **T** **F** **Q** **Q** | **G** **L** **H** **E** **F** **L** **K** **W** **D** **S** |
|
| 601 | 611 | 621 | 631 | 641 |
| **K** **I** **L** **D** **W** **M** **S** **V** **G** **L** | **S** **A** **L** **N** **G** **I** **P** **V** **I** **G** | **E** **I** **A** **D** **A** **A** **S** **I** **A** **L** | **E** **S** **E** **A** **Q** **A** **A** **Q** **V** **V** | **D** **T** **A** **I** **Q** **G** **G** **D** **M** **S** |
|
| 651 | 661 | 671 | 681 | 691 |
| **L** **A** **L** **K** **L** **A** **G** **I** **N** **M** | **A** **G** **A** **V** **V** **G** **A** **V** **G** **G** | **P** **T** **A** **R** **I** **G** **A** **K** **G** **A** | **A** **K** **G** **V** **A** **E** **V** **A** **A** **K** | **E** **A** **A** **E** **G** **A** **A** **K** **G** **T** |
|
| 701 | 711 | 721 | 731 | 741 |
| **A** **K** **G** **A** **A** **K** **G** **A** **G** **K** | **T** **A** **A** **E** **R** **P** **S** **A** **A** **E** | **F** **A** **K** **G** **Y** **V** **A** **G** **S** **N** | **I** **S** **K** **S** **T** **E** **I** **L** **K** **T** | **P** **V** **M** **A** **G** **L** **H** **Y** **E** **E** |
|
| 751 | 761 | 771 | 781 | 791 |
| **Y** **Q** **L** **D** **K** **Q** **K** **D** **G** **E** | **I** **H** **Q** **K** **L** **E** **H** **A** **G** **G** | **V** **P** **V** **G** **K** **Q** **I** **I** **P** **K** | **G** **I** **A** **D** **N** **F** **E** **G** **D** **V** | **R** **Q** **N** **L** **R** **N** **V** **R** **I** **R** |
|
| 801 |
| **R** **R** |

  
  
  
**Legend:  
  
The selection scale:**  

|  |
| --- |
| 1  2  3  4  5  6  7 |
| Positive selection           Purifying selection |
