# Supplementary material for: Acquisition and Evolution of Plant Pathogenesis–Associated Gene Clusters and Candidate Determinants of Tissue-Specificity in Xanthomonas
Source: PLoS One. 2008 Nov 27;3(11):e3828. doi: 10.1371/journal.pone.0003828 (PMC2585010; doi:10.1371/journal.pone.0003828)
Supplement: Data S1 — Selecton results. (0.10 MB ZIP) [file pone.0003828.s004.zip › Supplementary data - Selecton results/xcs_ver04.html]

Selecton Results: 1207255929

# Selecton Results

  
  

|
|  |
| 1 | 11 | 21 | 31 | 41 |
| **L** **Q** **R** **A** **W** **P** **S** **L** **N** **S** | **L** **L** **L** **A** **P** **G** **V** **R** **T** **A** | **V** **A** **G** **G** **V** **A** **A** **L** **L** **V** | **L** **Q** **G** **V** **R** **L** **L** **W** **L** **V** | **L** **T** **P** **I** **G** **P** **L** **G** **T** **A** |
|
| 51 | 61 | 71 | 81 | 91 |
| **Q** **A** **G** **A** **V** **A** **T** **L** **D** **V** | **S** **A** **L** **R** **R** **D** **V** **F** **Y** **R** | **S** **A** **S** **D** **A** **N** **R** **D** **G** **I** | **V** **L** **H** **G** **V** **R** **V** **G** **G** **T** | **Q** **A** **A** **A** **Y** **L** **S** **G** **S** **D** |
|
| 101 | 111 | 121 | 131 | 141 |
| **G** **R** **Q** **G** **A** **Y** **R** **V** **G** **D** | **T** **V** **A** **P** **G** **L** **M** **V** **Q** **A** | **I** **A** **A** **D** **H** **V** **L** **L** **R** **A** | **G** **G** **S** **V** **R** **R** **I** **A** **L** **S** | **E** **A** **S** **A** **A** **G** **A** **A** **P** **P** |
|
| 151 | 161 | 171 | 181 | 191 |
| **A** **A** **A** **T** **S** **A** **T** **A** **P** **A** | **I** **A** **P** **A** **A** **A** **Q** **S** **N** **V** | **A** **T** **A** **A** **D** **P** **T** **A** **A** **T** | **A** **V** **D** **P** **Q** **Q** **L** **L** **A** **S** | **A** **G** **L** **R** **A** **S** **A** **E** **G** **G** |
|
| 201 | 211 | 221 | 231 | 241 |
| **G** **F** **T** **L** **M** **P** **R** **G** **D** **G** | **A** **L** **L** **R** **Q** **A** **G** **L** **A** **P** | **G** **D** **V** **L** **T** **Q** **I** **N** **G** **R** | **T** **L** **D** **A** **E** **H** **L** **R** **E** **L** | **Q** **D** **E** **L** **R** **D** **G** **Q** **S** **A** |
|
| 251 | 261 | 271 | 281 | 291 |
| **T** **L** **T** **Y** **R** **R** **D** **G** **Q** **T** | **H** **T** **M** **T** **L** **K** **R** **P** **Q** **X** | **X** **X** **X** **X** **X** **X** **X** **X** **X** **X** | **X** **X** **X** **X** **X** **X** **X** **X** **X** **X** | **X** **X** **X** **X** **X** **X** **X** **X** **X** **X** |
|
| 301 | 311 | 321 | 331 | 341 |
| **X** **X** **M** **T** **V** **V** **R** **P** **L** **W** | **L** **I** **S** **A** **T** **L** **L** **L** **A** **L** | **P** **A** **V** **P** **M** **T** **A** **L** **H** **A** | **A** **D** **A** **P** **A** **V** **R** **L** **Q** **D** | **V** **D** **L** **R** **A** **F** **I** **Q** **D** **V** |
|
| 351 | 361 | 371 | 381 | 391 |
| **S** **R** **A** **T** **G** **I** **T** **F** **I** **V** | **D** **T** **R** **V** **Q** **G** **S** **V** **N** **V** | **A** **R** **A** **Q** **A** **M** **S** **E** **A** **D** | **L** **L** **G** **M** **L** **L** **A** **V** **L** **R** | **A** **N** **G** **L** **I** **A** **V** **S** **S** **G** |
|
| 401 | 411 | 421 | 431 | 441 |
| **P** **S** **T** **Y** **R** **V** **I** **P** **D** **D** | **T** **A** **A** **Q** **Q** **P** **G** **S** **A** **A** | **N** **G** **N** **L** **G** **F** **A** **T** **Q** **V** | **F** **T** **L** **Q** **R** **V** **D** **A** **R** **S** | **A** **A** **E** **I** **L** **K** **P** **L** **I** **G** |
|
| 451 | 461 | 471 | 481 | 491 |
| **R** **G** **G** **V** **I** **M** **A** **M** **P** **Q** | **G** **N** **S** **L** **L** **I** **A** **D** **Y** **A** | **D** **N** **L** **R** **R** **I** **R** **T** **L** **V** | **A** **Q** **I** **D** **T** **D** **R** **A** **A** **I** | **D** **T** **V** **T** **L** **R** **N** **S** **S** **A** |
|
| 501 | 511 | 521 | 531 | 541 |
| **Q** **E** **L** **A** **R** **T** **L** **T** **S** **L** | **F** **G** **Q** **G** **G** **E** **R** **S** **N** **V** | **L** **S** **V** **L** **P** **V** **D** **S** **S** **N** | **S** **L** **I** **V** **R** **G** **D** **P** **A** **L** | **V** **Q** **R** **V** **V** **R** **T** **A** **V** **D** |
|
| 551 | 561 | 571 | 581 | 591 |
| **L** **D** **G** **R** **A** **E** **R** **R** **G** **D** | **V** **S** **V** **V** **R** **L** **Q** **H** **A** **S** | **A** **E** **Q** **L** **L** **P** **V** **L** **Q** **Q** | **L** **V** **G** **Q** **T** **P** **G** **N** **E** **A** | **Q** **V** **G** **Q** **D** **T** **R** **L** **A** **T** |
|
| 601 | 611 | 621 | 631 | 641 |
| **I** **D** **V** **A** **A** **A** **S** **G** **A** **A** | **Q** **T** **Q** **V** **I** **A** **P** **A** **A** **G** | **K** **R** **P** **V** **I** **V** **R** **Y** **P** **G** | **S** **N** **A** **L** **I** **I** **N** **A** **D** **P** | **E** **T** **Q** **R** **A** **L** **M** **D** **V** **I** |
|
| 651 | 661 | 671 | 681 | 691 |
| **R** **Q** **L** **D** **V** **H** **R** **E** **Q** **V** | **L** **V** **E** **A** **I** **V** **V** **E** **I** **S** | **D** **T** **A** **A** **K** **R** **L** **G** **V** **Q** | **L** **L** **L** **A** **G** **R** **N** **G** **T** **V** | **P** **L** **V** **A** **T** **Q** **Y** **S** **G** **A** |
|
| 701 | 711 | 721 | 731 | 741 |
| **S** **P** **G** **I** **V** **P** **L** **A** **A** **A** | **A** **A** **G** **T** **R** **S** **G** **N** **A** **D** | **D** **D** **S** **V** **L** **E** **Q** **A** **R** **N** | **V** **A** **A** **Q** **S** **L** **L** **G** **L** **S** | **G** **G** **L** **I** **G** **L** **A** **G** **Q** **S** |
|
| 751 | 761 | 771 | 781 | 791 |
| **N** **D** **A** **V** **F** **G** **M** **I** **I** **D** | **A** **V** **K** **S** **D** **T** **G** **S** **N** **L** | **L** **S** **T** **P** **S** **I** **M** **T** **L** **D** | **N** **E** **Q** **A** **R** **I** **L** **V** **G** **Q** | **E** **V** **P** **I** **T** **T** **G** **E** **V** **L** |
|
| 801 | 811 | 821 | 831 | 841 |
| **G** **A** **A** **N** **D** **N** **P** **F** **R** **T** | **I** **Q** **R** **Q** **D** **V** **G** **V** **E** **L** | **E** **V** **R** **P** **Q** **I** **N** **T** **A** **G** | **G** **I** **T** **L** **A** **I** **K** **Q** **E** **V** | **S** **A** **I** **A** **G** **P** **V** **S** **A** **Q** |
|
| 851 | 861 | 871 | 881 | 891 |
| **S** **S** **E** **L** **V** **F** **N** **K** **R** **Q** | **I** **E** **T** **R** **V** **V** **V** **E** **N** **G** | **A** **I** **V** **A** **L** **G** **G** **L** **L** **D** | **Q** **N** **D** **R** **Q** **T** **V** **E** **K** **V** | **P** **L** **L** **G** **D** **V** **P** **G** **L** **G** |
|
| 901 | 911 | 921 | 931 | 941 |
| **A** **L** **F** **R** **H** **K** **S** **R** **N** **R** | **D** **K** **T** **N** **L** **M** **V** **F** **I** **R** | **P** **T** **I** **I** **R** **D** **A** **A** **D** **A** | **Q** **R** **M** **T** **A** **P** **R** **Y** **T** **Y** | **L** **R** **E** **R** **Q** **L** **A** **D** **G** **D** |
|
| 951 | 961 | 971 | 981 | 991 |
| **P** **E** **A** **A** **L** **D** **A** **L** **V** **R** | **D** **Y** **L** **R** **A** **Q** **P** **P** **Q** **L** | **P** **A** **R** **P** **S** **S** **V** **A** **P** **A** | **P** **A** **T** **T** **P** **A** **P** **G** **A** **R** | **P** **V** **Q** **R** **X** **X** **X** **X** **X** **X** |
|
| 1001 | 1011 | 1021 | 1031 | 1041 |
| **X** **X** **X** **X** **X** **X** **X** **X** **X** **X** | **X** **X** **X** **X** **X** **X** **X** **X** **X** **X** | **X** **X** **X** **X** **X** **X** **X** **M** **S** **T** | **A** **R** **A** **G** **I** **S** **Y** **A** **F** **A** | **K** **R** **H** **G** **V** **V** **L** **L** **G** **S** |
|
| 1051 | 1061 | 1071 | 1081 | 1091 |
| **D** **T** **T** **A** **Q** **I** **G** **L** **R** **E** | **G** **G** **D** **V** **Q** **A** **L** **I** **E** **L** | **R** **R** **A** **L** **G** **L** **P** **L** **Q** **V** | **R** **T** **L** **A** **P** **S** **V** **F** **D** **R** | **H** **V** **S** **E** **I** **Y** **A** **D** **A** **G** |
|
| 1101 | 1111 | 1121 | 1131 | 1141 |
| **L** **E** **Q** **G** **V** **Q** **V** **E** **A** **L** | **D** **L** **H** **G** **S** **L** **D** **S** **L** **I** | **D** **D** **I** **P** **T** **A** **D** **L** **L** **D** | **S** **Q** **D** **D** **A** **P** **I** **I** **R** **L** | **I** **N** **G** **I** **I** **A** **E** **A** **A** **R** |
|
| 1151 | 1161 | 1171 | 1181 | 1191 |
| **L** **G** **A** **S** **D** **V** **H** **L** **E** **S** | **Y** **E** **S** **R** **L** **R** **V** **R** **L** **R** | **V** **D** **G** **V** **M** **R** **E** **A** **A** **T** | **L** **P** **G** **R** **I** **A** **P** **L** **L** **V** | **S** **R** **V** **K** **V** **M** **A** **R** **L** **D** |
|
| 1201 | 1211 | 1221 | 1231 | 1241 |
| **I** **A** **E** **K** **R** **I** **P** **Q** **D** **G** | **R** **V** **S** **L** **V** **M** **G** **A** **K** **A** | **L** **D** **V** **R** **V** **S** **T** **L** **P** **T** | **R** **G** **S** **E** **R** **V** **V** **L** **R** **I** | **L** **D** **K** **E** **Q** **G** **T** **L** **S** **L** |
|
| 1251 | 1261 | 1271 | 1281 | 1291 |
| **A** **Q** **L** **G** **M** **P** **P** **A** **V** **M** | **H** **T** **V** **Q** **R** **A** **L** **Q** **V** **P** | **N** **G** **I** **V** **L** **V** **T** **G** **P** **T** | **G** **S** **G** **K** **T** **T** **T** **L** **Y** **A** | **A** **L** **R** **L** **L** **N** **D** **G** **S** **R** |
|
| 1301 | 1311 | 1321 | 1331 | 1341 |
| **N** **I** **L** **T** **V** **E** **D** **P** **V** **E** | **Y** **A** **I** **D** **G** **V** **G** **Q** **T** **Q** | **V** **N** **A** **R** **V** **G** **M** **T** **F** **A** | **A** **G** **L** **R** **A** **I** **L** **R** **Q** **D** | **P** **D** **V** **V** **M** **I** **G** **E** **I** **R** |
|
| 1351 | 1361 | 1371 | 1381 | 1391 |
| **D** **T** **E** **T** **A** **Q** **I** **A** **V** **Q** | **A** **S** **L** **T** **G** **H** **L** **V** **L** **S** | **T** **V** **H** **T** **N** **D** **A** **V** **G** **A** | **V** **T** **R** **L** **R** **D** **M** **G** **I** **E** | **P** **F** **L** **L** **A** **S** **S** **L** **R** **L** |
|
| 1401 | 1411 | 1421 | 1431 | 1441 |
| **I** **L** **A** **Q** **R** **L** **V** **R** **R** **L** | **C** **T** **Q** **C** **R** **S** **A** **R** **P** **M** | **D** **A** **G** **T** **A** **H** **L** **L** **R** **A** | **P** **A** **D** **A** **V** **I** **Y** **A** **A** **A** | **G** **C** **N** **A** **C** **H** **H** **S** **G** **Y** |
|
| 1451 | 1461 | 1471 | 1481 | 1491 |
| **A** **G** **R** **V** **G** **I** **Y** **E** **A** **I** | **A** **V** **D** **D** **A** **M** **R** **R** **L** **I** | **G** **D** **N** **A** **D** **E** **D** **A** **L** **A** | **A** **V** **A** **F** **A** **R** **V** **P** **R** **L** | **G** **E** **A** **A** **R** **A** **A** **V** **L** **Q** |
|
| 1501 | 1511 | 1521 | 1531 | 1541 |
| **G** **L** **T** **T** **L** **E** **E** **A** **L** **R** | **V** **T** **R** **Q** **Q** **D** **D** **A** **H** **A** | **A** **V** **X** **X** **X** **X** **X** **X** **X** **X** | **X** **X** **X** **X** **X** **X** **X** **X** **X** **X** | **X** **X** **X** **X** **X** **X** **X** **X** **X** **X** |
|
| 1551 | 1561 | 1571 | 1581 | 1591 |
| **X** **X** **X** **X** **X** **M** **R** **M** **P** **Q** | **F** **D** **Y** **T** **V** **L** **D** **L** **H** **G** | **R** **N** **R** **H** **G** **V** **I** **S** **A** **D** | **S** **I** **N** **S** **A** **R** **A** **Q** **L** **E** | **Q** **R** **Q** **W** **V** **P** **V** **R** **V** **E** |
|
| 1601 | 1611 | 1621 | 1631 | 1641 |
| **V** **A** **A** **A** **T** **A** **S** **T** **A** **V** | **R** **A** **A** **R** **F** **S** **G** **K** **D** **L** | **V** **L** **F** **T** **R** **Q** **L** **A** **T** **L** | **V** **E** **T** **A** **P** **L** **E** **E** **A** **L** | **R** **T** **I** **G** **T** **Q** **S** **E** **R** **R** |
|
| 1651 | 1661 | 1671 | 1681 | 1691 |
| **G** **V** **R** **R** **V** **T** **S** **Q** **T** **H** | **A** **L** **V** **V** **E** **G** **F** **R** **L** **S** | **D** **A** **M** **A** **R** **Q** **G** **K** **A** **F** | **P** **A** **L** **Y** **R** **A** **M** **V** **A** **A** | **G** **E** **S** **A** **G** **A** **L** **P** **Q** **V** |
|
| 1701 | 1711 | 1721 | 1731 | 1741 |
| **L** **E** **R** **L** **A** **D** **L** **L** **E** **R** | **Q** **A** **Q** **V** **R** **S** **K** **L** **Q** **S** | **A** **L** **V** **Y** **P** **T** **A** **L** **A** **A** | **T** **A** **G** **A** **V** **V** **I** **V** **L** **M** | **T** **F** **V** **V** **P** **K** **V** **V** **D** **Q** |
|
| 1751 | 1761 | 1771 | 1781 | 1791 |
| **F** **D** **S** **M** **G** **R** **A** **L** **P** **W** | **L** **T** **R** **V** **V** **I** **G** **V** **S** **H** | **F** **L** **L** **H** **A** **G** **I** **P** **L** **L** | **I** **A** **L** **V** **V** **A** **L** **V** **A** **A** | **V** **R** **L** **L** **K** **R** **P** **A** **L** **R** |
|
| 1801 | 1811 | 1821 | 1831 | 1841 |
| **L** **A** **A** **D** **R** **A** **L** **L** **R** **A** | **P** **L** **L** **G** **R** **L** **I** **R** **D** **L** | **H** **A** **A** **R** **M** **A** **R** **T** **L** **A** | **I** **M** **V** **N** **S** **G** **L** **P** **L** **M** | **E** **G** **L** **M** **I** **A** **A** **R** **T** **V** |
|
| 1851 | 1861 | 1871 | 1881 | 1891 |
| **D** **N** **R** **A** **L** **R** **L** **A** **T** **D** | **S** **M** **V** **T** **A** **I** **R** **E** **G** **G** | **S** **L** **A** **A** **A** **M** **K** **R** **A** **G** | **V** **F** **P** **P** **T** **L** **L** **Y** **M** **A** | **S** **S** **G** **E** **N** **S** **G** **R** **L** **A** |
|
| 1901 | 1911 | 1921 | 1931 | 1941 |
| **P** **M** **L** **E** **R** **A** **A** **D** **Y** **L** | **E** **R** **E** **F** **E** **A** **F** **T** **T** **A** | **A** **M** **S** **L** **L** **E** **P** **A** **I** **I** | **V** **L** **L** **G** **G** **V** **V** **A** **V** **I** | **V** **L** **S** **I** **L** **L** **P** **I** **L** **Q** |
|
| 1951 | 1961 | 1971 | 1981 | 1991 |
| **F** **N** **T** **L** **A** **L** **G** **X** **X** **X** | **X** **X** **X** **X** **X** **X** **X** **X** **X** **X** | **X** **X** **X** **X** **X** **X** **X** **X** **X** **X** | **X** **X** **X** **X** **X** **X** **X** **X** **X** **X** | **M** **Q** **S** **I** **H** **S** **R** **A** **R** **L** |
|
| 2001 | 2011 | 2021 | 2031 | 2041 |
| **G** **S** **P** **T** **A** **Q** **R** **R** **T** **R** | **G** **F** **T** **L** **V** **E** **L** **M** **V** **V** | **I** **V** **I** **I** **G** **L** **L** **A** **T** **V** | **V** **M** **I** **N** **V** **M** **P** **S** **Q** **D** | **R** **A** **M** **M** **E** **K** **A** **R** **A** **D** |
|
| 2051 | 2061 | 2071 | 2081 | 2091 |
| **V** **A** **V** **L** **E** **Q** **A** **L** **E** **T** | **Y** **R** **L** **D** **N** **L** **S** **Y** **P** **S** | **T** **E** **Q** **G** **L** **Q** **A** **L** **L** **D** | **P** **P** **S** **G** **L** **T** **R** **P** **E** **R** | **Y** **R** **H** **G** **G** **Y** **I** **R** **R** **L** |
|
| 2101 | 2111 | 2121 | 2131 | 2141 |
| **P** **E** **D** **P** **W** **G** **H** **A** **Y** **Q** | **Y** **R** **R** **P** **G** **R** **N** **G** **G** **F** | **D** **V** **Y** **S** **L** **G** **A** **D** **G** **A** | **E** **G** **G** **D** **A** **D** **N** **A** **D** **I** | **G** **N** **W** **R** **X** **X** **X** **X** **X** **X** |
|
| 2151 | 2161 | 2171 | 2181 | 2191 |
| **X** **X** **X** **X** **X** **X** **X** **X** **X** **X** | **X** **X** **X** **X** **X** **X** **X** **X** **X** **X** | **X** **X** **X** **X** **X** **X** **X** **M** **R** **M** | **H** **V** **A** **P** **M** **R** **A** **R** **G** **F** | **T** **L** **L** **E** **V** **L** **A** **V** **L** **V** |
|
| 2201 | 2211 | 2221 | 2231 | 2241 |
| **I** **T** **A** **L** **A** **S** **T** **L** **V** **V** | **M** **T** **L** **P** **D** **T** **R** **R** **D** **L** | **H** **D** **H** **A** **D** **T** **L** **A** **S** **A** | **L** **M** **H** **A** **R** **D** **E** **A** **I** **M** | **S** **L** **R** **M** **V** **E** **V** **S** **I** **D** |
|
| 2251 | 2261 | 2271 | 2281 | 2291 |
| **A** **G** **G** **Y** **A** **F** **R** **R** **Q** **A** | **Q** **Q** **R** **W** **I** **A** **L** **D** **E** **T** | **P** **F** **A** **T** **M** **R** **W** **P** **E** **G** | **I** **Q** **A** **Q** **L** **P** **A** **G** **S** **K** | **Q** **L** **S** **V** **R** **F** **D** **P** **T** **G** |
|
| 2301 | 2311 | 2321 | 2331 | 2341 |
| **A** **A** **T** **P** **Q** **R** **V** **A** **L** **G** | **D** **G** **R** **Q** **Q** **M** **Q** **I** **V** **V** | **D** **A** **A** **G** **V** **V** **Q** **V** **D** **A** | **P** **S** **R** **X** **X** **X** **X** **X** **X** **X** | **X** **X** **X** **X** **X** **X** **X** **X** **X** **X** |
|
| 2351 | 2361 | 2371 | 2381 | 2391 |
| **X** **X** **X** **X** **X** **X** **X** **X** **X** **X** | **X** **X** **X** **X** **X** **X** **M** **R** **P** **L** | **A** **D** **A** **R** **A** **S** **G** **G** **F** **S** | **L** **L** **E** **L** **M** **V** **A** **L** **A** **I** | **F** **G** **M** **A** **V** **V** **G** **L** **L** **N** |
|
| 2401 | 2411 | 2421 | 2431 | 2441 |
| **L** **S** **G** **E** **S** **A** **R** **T** **A** **M** | **V** **L** **E** **E** **R** **A** **L** **A** **A** **V** | **V** **A** **E** **N** **Q** **A** **I** **E** **A** **T** | **L** **A** **P** **A** **S** **A** **I** **R** **A** **P** | **A** **N** **G** **Q** **V** **M** **L** **G** **G** **R** |
|
| 2451 | 2461 | 2471 | 2481 | 2491 |
| **S** **W** **E** **W** **Q** **R** **Q** **S** **L** **P** | **A** **G** **A** **A** **G** **L** **L** **R** **I** **E** | **V** **R** **V** **R** **A** **A** **G** **Q** **M** **Q** | **E** **V** **A** **S** **L** **S** **M** **L** **R** **S** | **A** **E** **X** **X** **X** **X** **X** **X** **X** **X** |
|
| 2501 | 2511 | 2521 | 2531 | 2541 |
| **X** **X** **X** **X** **X** **X** **X** **X** **X** **X** | **X** **X** **X** **X** **X** **X** **X** **X** **X** **X** | **X** **X** **X** **X** **X** **M** **I** **R** **K** **Q** | **R** **T** **R** **G** **F** **T** **L** **I** **E** **L** | **L** **V** **A** **L** **A** **V** **F** **A** **L** **V** |
|
| 2551 | 2561 | 2571 | 2581 | 2591 |
| **A** **A** **A** **A** **V** **A** **V** **M** **R** **Q** | **S** **I** **D** **Q** **R** **D** **A** **V** **R** **A** | **R** **L** **Q** **Q** **I** **R** **D** **F** **Q** **L** | **A** **H** **G** **L** **L** **R** **S** **D** **L** **Q** | **Q** **A** **A** **V** **R** **R** **T** **R** **N** **S** |
|
| 2601 | 2611 | 2621 | 2631 | 2641 |
| **E** **G** **S** **A** **A** **R** **S** **A** **F** **V** | **A** **S** **A** **S** **G** **T** **Y** **G** **P** **L** | **F** **G** **F** **V** **R** **R** **G** **W** **S** **N** | **P** **D** **Q** **A** **P** **R** **A** **S** **L** **Q** | **Y** **V** **E** **Y** **R** **I** **V** **D** **G** **R** |
|
| 2651 | 2661 | 2671 | 2681 | 2691 |
| **L** **E** **R** **S** **A** **R** **S** **A** **L** **D** | **G** **G** **A** **A** **G** **T** **P** **Q** **V** **L** | **L** **R** **G** **V** **R** **S** **A** **A** **V** **A** | **F** **H** **Y** **R** **A** **Q** **W** **S** **D** **G** | **W** **S** **G** **G** **L** **E** **A** **L** **P** **D** |
|
| 2701 | 2711 | 2721 | 2731 | 2741 |
| **A** **V** **A** **L** **E** **L** **D** **L** **E** **H** | **W** **G** **R** **V** **R** **Q** **L** **F** **L** **L** | **P** **E** **G** **R** **T** **X** **X** **X** **X** **X** | **X** **X** **X** **X** **X** **X** **X** **X** **X** **X** | **X** **X** **X** **X** **X** **X** **X** **X** **X** **X** |
|
| 2751 | 2761 | 2771 | 2781 | 2791 |
| **X** **X** **X** **X** **X** **X** **X** **X** **M** **A** | **L** **H** **A** **P** **A** **R** **Q** **R** **G** **V** | **A** **L** **L** **T** **V** **L** **L** **L** **V** **A** | **V** **M** **T** **L** **L** **M** **A** **A** **V** **L** | **E** **D** **L** **R** **F** **G** **L** **R** **R** **S** |
|
| 2801 | 2811 | 2821 | 2831 | 2841 |
| **G** **N** **G** **E** **A** **M** **T** **Q** **A** **Q** | **W** **Y** **A** **L** **G** **S** **E** **A** **V** **A** | **R** **Q** **R** **L** **Q** **A** **L** **S** **E** **R** | **D** **P** **L** **R** **T** **T** **L** **D** **G** **G** | **W** **N** **D** **Q** **P** **I** **G** **F** **P** **L** |
|
| 2851 | 2861 | 2871 | 2881 | 2891 |
| **D** **D** **G** **A** **V** **S** **V** **R** **L** **H** | **D** **R** **G** **G** **C** **F** **N** **L** **N** **S** | **V** **V** **V** **G** **A** **P** **E** **Q** **W** **Q** | **R** **S** **D** **E** **G** **A** **R** **Q** **Y** **R** | **A** **L** **L** **D** **V** **L** **G** **I** **A** **P** |
|
| 2901 | 2911 | 2921 | 2931 | 2941 |
| **Q** **Q** **A** **H** **A** **L** **T** **D** **A** **L** | **V** **D** **W** **I** **D** **S** **D** **T** **Q** **P** | **G** **V** **H** **G** **A** **E** **D** **D** **R** **Y** | **L** **Q** **A** **A** **A** **P** **Q** **R** **T** **G** | **A** **T** **L** **L** **A** **G** **V** **S** **E** **L** |
|
| 2951 | 2961 | 2971 | 2981 | 2991 |
| **R** **A** **I** **A** **G** **Y** **T** **P** **Q** **L** | **I** **A** **Q** **L** **Q** **P** **Y** **V** **C** **A** | **L** **P** **E** **G** **R** **L** **S** **P** **I** **N** | **I** **N** **T** **L** **R** **L** **D** **D** **A** **P** | **V** **L** **V** **A** **L** **T** **G** **G** **A** **L** |
|
| 3001 | 3011 | 3021 | 3031 | 3041 |
| **E** **L** **P** **A** **A** **R** **R** **V** **I** **A** | **A** **R** **P** **A** **G** **G** **W** **R** **D** **I** | **R** **M** **F** **L** **S** **Q** **P** **A** **L** **M** | **Q** **A** **E** **L** **S** **N** **A** **V** **M** **E** | **Q** **L** **A** **L** **R** **T** **R** **Y** **F** **S** |
|
| 3051 | 3061 | 3071 | 3081 | 3091 |
| **L** **Y** **S** **Q** **V** **D** **H** **A** **G** **A** | **Q** **V** **M** **L** **E** **A** **L** **L** **Q** **Q** | **E** **T** **A** **G** **R** **I** **R** **L** **V** **A** | **R** **Q** **W** **S** **S** **D** **Q** **X** **X** **X** | **X** **X** **X** **X** **X** **X** **X** **X** **X** **X** |
|
| 3101 | 3111 | 3121 | 3131 | 3141 |
| **X** **X** **X** **X** **X** **X** **X** **X** **X** **X** | **X** **X** **X** **X** **X** **X** **X** **X** **X** **X** | **M** **T** **T** **T** **L** **L** **L** **L** **P** **A** | **D** **A** **A** **A** **E** **S** **I** **A** **V** **R** | **A** **D** **A** **Q** **G** **Q** **V** **Q** **S** **V** |
|
| 3151 | 3161 | 3171 | 3181 | 3191 |
| **G** **H** **A** **T** **A** **P** **P** **E** **P** **S** | **A** **R** **A** **L** **L** **V** **V** **P** **G** **V** | **D** **V** **H** **L** **R** **W** **L** **R** **L** **P** | **G** **R** **S** **M** **A** **Q** **S** **R** **A** **A** | **A** **R** **L** **Q** **L** **A** **E** **H** **L** **A** |
|
| 3201 | 3211 | 3221 | 3231 | 3241 |
| **I** **E** **T** **Q** **A** **L** **H** **V** **V** **I** | **A** **G** **S** **A** **E** **A** **D** **G** **T** **R** | **L** **V** **A** **A** **V** **D** **S** **V** **V** **M** | **Q** **Q** **W** **L** **A** **R** **A** **T** **Q** **L** | **G** **F** **A** **P** **D** **A** **V** **V** **P** **D** |
|
| 3251 | 3261 | 3271 | 3281 | 3291 |
| **C** **L** **L** **L** **E** **P** **G** **A** **D** **A** | **T** **A** **A** **V** **M** **H** **W** **D** **G** **R** | **W** **L** **I** **R** **G** **A** **Q** **L** **A** **C** | **S** **L** **E** **P** **E** **A** **A** **R** **L** **L** | **L** **G** **D** **R** **V** **P** **A** **V** **P** **P** |
|
| 3301 | 3311 | 3321 | 3331 | 3341 |
| **G** **P** **D** **P** **T** **H** **S** **I** **A** **G** | **F** **A** **R** **S** **A** **A** **H** **V** **P** **I** | **D** **L** **L** **Q** **H** **P** **F** **A** **N** **K** | **A** **A** **A** **E** **Q** **K** **R** **A** **P** **R** | **R** **L** **A** **V** **L** **A** **A** **L** **V** **L** |
|
| 3351 | 3361 | 3371 | 3381 | 3391 |
| **L** **S** **P** **V** **L** **L** **L** **L** **A** **Q** | **T** **L** **R** **Y** **E** **I** **G** **A** **R** **V** | **L** **Q** **A** **R** **T** **A** **T** **Q** **L** **G** | **V** **H** **D** **A** **A** **A** **V** **P** **A** **A** | **L** **Q** **A** **R** **R** **Q** **A** **G** **T** **A** |
|
| 3401 | 3411 | 3421 | 3431 | 3441 |
| **T** **D** **R** **L** **A** **M** **Q** **L** **S** **T** | **L** **F** **A** **A** **V** **D** **T** **L** **P** **A** | **A** **E** **L** **D** **Q** **L** **D** **Y** **N** **P** | **A** **Q** **P** **L** **R** **A** **T** **L** **L** **H** | **T** **D** **D** **A** **S** **L** **Q** **Q** **L** **S** |
|
| 3451 | 3461 | 3471 | 3481 | 3491 |
| **A** **R** **L** **A** **D** **A** **G** **W** **H** **L** | **D** **P** **G** **S** **S** **Q** **V** **E** **D** **G** | **R** **M** **R** **T** **P** **F** **T** **L** **E** **P** | **L** **R** **X** **X** **X** **X** **X** **X** **X** **X** | **X** **X** **X** **X** **X** **X** **X** **X** **X** **X** |
|
| 3501 | 3511 | 3521 | 3531 | 3541 |
| **X** **X** **X** **X** **X** **X** **X** **X** **X** **X** | **X** **X** **X** **X** **X** **M** **K** **A** **S** **L** | **Q** **R** **G** **T** **Q** **W** **W** **Q** **T** **R** | **A** **P** **R** **E** **R** **R** **M** **L** **G** **V** | **M** **C** **A** **A** **L** **A** **A** **F** **V** **G** |
|
| 3551 | 3561 | 3571 | 3581 | 3591 |
| **W** **Y** **A** **L** **Y** **V** **P** **L** **R** **H** | **W** **H** **D** **R** **A** **W** **A** **R** **Y** **A** | **D** **A** **A** **Q** **L** **A** **L** **D** **A** **S** | **A** **Q** **A** **A** **S** **T** **R** **R** **A** **A** | **A** **L** **G** **S** **A** **A** **L** **A** **D** **I** |
|
| 3601 | 3611 | 3621 | 3631 | 3641 |
| **I** **A** **S** **S** **A** **R** **D** **A** **G** **V** | **N** **I** **T** **S** **Q** **Q** **R** **S** **A** **V** | **G** **G** **V** **D** **V** **Q** **I** **E** **A** **V** | **S** **A** **A** **A** **L** **F** **G** **W** **L** **E** | **R** **L** **R** **Q** **A** **H** **G** **L** **A** **P** |
|
| 3651 | 3661 | 3671 | 3681 | 3691 |
| **T** **Q** **L** **S** **V** **A** **R** **E** **Q** **G** | **Q** **L** **R** **V** **R** **C** **G** **F** **A** **E** | **S** **G** **P** **X** **X** **X** **X** **X** **X** **X** | **X** **X** **X** **X** **X** **X** **X** **X** **X** **X** | **X** **X** **X** **X** **X** **X** **X** **X** **X** **X** |
|
| 3701 | 3711 | 3721 | 3731 | 3741 |
| **X** **X** **X** **X** **X** **X** **V** **T** **R** **M** | **R** **W** **A** **L** **I** **L** **V** **S** **M** **A** | **G** **V** **A** **L** **L** **A** **F** **V** **P** **L** | **R** **L** **V** **L** **P** **R** **E** **G** **L** **P** | **F** **S** **V** **L** **D** **V** **E** **G** **P** **I** |
|
| 3751 | 3761 | 3771 | 3781 | 3791 |
| **W** **A** **G** **T** **L** **R** **Q** **V** **Q** **W** | **Q** **G** **I** **V** **F** **G** **D** **V** **A** **V** | **R** **P** **Q** **V** **W** **P** **L** **L** **R** **G** | **E** **R** **R** **V** **Q** **L** **Q** **S** **T** **Q** | **L** **Q** **L** **L** **L** **V** **D** **G** **A** **Q** |
|
| 3801 | 3811 | 3821 | 3831 | 3841 |
| **R** **G** **M** **R** **N** **A** **Q** **G** **Q** **L** | **L** **V** **R** **Q** **P** **G** **G** **V** **A** **L** | **I** **D** **L** **A** **V** **Q** **L** **Q** **Q** **V** | **D** **L** **L** **F** **D** **A** **T** **G** **C** **V** | **Q** **A** **H** **G** **R** **V** **S** **L** **Q** **L** |
|
| 3851 | 3861 | 3871 | 3881 | 3891 |
| **L** **A** **S** **G** **A** **G** **A** **L** **P** **G** | **L** **P** **P** **L** **R** **L** **S** **G** **Q** **P** | **A** **C** **V** **D** **H** **T** **A** **V** **L** **T** | **L** **L** **P** **D** **T** **A** **L** **P** **A** **G** | **V** **Q** **A** **K** **A** **Q** **L** **Q** **L** **W** |
|
| 3901 | 3911 | 3921 | 3931 | 3941 |
| **P** **D** **G** **R** **W** **Q** **L** **Q** **S** **R** | **V** **D** **P** **A** **Q** **D** **A** **V** **L** **G** | **V** **G** **L** **Q** **L** **L** **G** **F** **A** **A** | **T** **P** **E** **R** **G** **F** **L** **R** **N** **D** | **E** **G** **R** **L** **H** |

  
  
  
**Legend:  
  
The selection scale:**  

|  |
| --- |
| 1  2  3  4  5  6  7 |
| Positive selection           Purifying selection |
